# Central limit theorems for sub-linear expectation under the Lindeberg condition

**DOI:** 10.1186/s13660-018-1901-x

**Published:** 2018-11-16

**Authors:** Cheng Hu

**Affiliations:** School of Mathematics and Statistics, Shandong Normal University, Jinan, China

**Keywords:** 60F05, Central limit theorem, Sub-linear expectation, Capacity, Lindeberg condition, G-normal distribution

## Abstract

In this paper, we investigate the central limit theorems for sub-linear expectation for a sequence of independent random variables without assumption of identical distribution. We first give a bound on the distance between the normalized sum distribution and G-normal distribution which can be used to derive the central limit theorem for sub-linear expectation under the Lindeberg condition. Then we obtain the central limit theorem for capacity under the Lindeberg condition. We also get the central limit theorem for capacity for summability methods under the Lindeberg condition.

## Introduction

Peng [15] put forward the theory of sub-linear expectation to describe the probability uncertainties in statistics and economics which are difficult to be handled by classical probability theory. There has been increasing interest in sub-linear expectation (see, for example, [1, 2, 4, 11, 18, 26]).

The classical central limit theorem (CLT for short) is a fundamental result in probability theory. Peng [16] initiated the CLT for sub-linear expectation for a sequence of i.i.d. random variables with finite $(2+\alpha)$-moments for some $\alpha >0$. The CLT for sub-linear expectation has gotten considerable development. Hu and Zhang [10] obtained a CLT for capacity. Li and Shi [13] got a CLT for sub-linear expectation without assumption of identical distribution. Hu [9] extended Peng’s CLT by weakening the assumptions of test functions. Zhang and Chen [21] derived a weighted CLT for sub-linear expectation. Hu and Zhou [12] presented some multi-dimensional CLTs without assumption of identical distribution. Li [14] proved a CLT for sub-linear expectation for a sequence of m-dependent random variables. Rokhlin [19] gave a CLT under the Lindeberg condition under classical probability with variance uncertainty. Zhang [22] gained a CLT for sub-linear expectation under a moment condition weaker than $(2+\alpha)$-moments. Zhang [23] established a martingale CLT and functional CLT for sub-linear expectation under the Lindeberg condition.

The purpose of this paper is to investigate the CLTs for sub-linear expectation for a sequence of independent random variables without assumption of identical distribution. We first give a bound on the distance between the normalized sum distribution $\mathbb{E}[\varphi (\frac{S_{n}}{\overline{B}_{n}})]$ and G-normal distribution $\widetilde{\mathbb{E}}[\varphi (\xi)]$, where $\xi \sim \mathcal{N}( \{0\};[\underline{\sigma }^{2},1])$. It can be used to derive the CLT for sub-linear expectation under the Lindeberg condition directly, which coincides with the result in Zhang [23]. Different from the classical case, when choosing $\underline{B}_{n}$ as the normalizing factor, we can also obtain a bound on the distance between the normalized sum distribution $\mathbb{E}[\varphi (\frac{S_{n}}{\underline{B}_{n}})]$ and the corresponding G-normal random variable $\mathbb{E}[\varphi ( \eta)]$ where $\eta \sim \mathcal{N}(\{0\};[1,\overline{\sigma }^{2}])$. Secondly, we obtain a CLT for capacity under the Lindeberg condition which extends the CLT for capacity for a sequence of i.i.d. random variables in Hu and Zhang [10]. We also study the CLT for capacity for summability methods under the Lindeberg condition. The regular summability method is an important subject in functional analysis. In recent years it has been found that summability method plays an important role in the study of statistical convergence (see [5–7, 20]). So it is meaningful to investigate the CLT for capacity for summability methods.

This paper is organized as follows. In Sect. 2, we recall some basic concepts and lemmas related to the main results. In Sect. 3, we give a bound on the distance between the normalized sum distribution and G-normal distribution. In Sect. 4, we prove a CLT for capacity under the Lindeberg condition. In Sect. 5, we show a CLT for capacity for summability methods under the Lindeberg condition.

## Basic concepts and lemmas

This paper is studied under the sub-linear expectation framework established by Peng [15–18]. Let $(\varOmega,\mathcal{F})$ be a given measurable space. Let $\mathcal{H}$ be a linear space of real functions defined on *Ω* such that if $X_{1},X_{2},\ldots,X_{n}\in \mathcal{H}$ then $\varphi (X_{1},X_{2},\ldots,X_{n})\in \mathcal{H}$ for each $\varphi \in C_{l,\mathrm{Lip}}(\mathbb{R}^{n})$ where $C_{l,\mathrm{Lip}}(\mathbb{R}^{n})$ denotes the linear space of local Lipschitz continuous functions *φ* satisfying

$$ \bigl\vert \varphi (\boldsymbol{x})-\varphi (\boldsymbol{y}) \bigr\vert \leq C\bigl(1+ \vert \boldsymbol{x} \vert ^{m}+ \vert \boldsymbol{y} \vert ^{m}\bigr) \vert \boldsymbol{x}- \boldsymbol{y} \vert , \quad \forall \boldsymbol{x},\boldsymbol{y}\in \mathbb{R} ^{n}, $$

for some $C>0$, $m\in \mathbb{N}$ depending on *φ*. $\mathcal{H}$ contains all $I_{A}$ where $A\in \mathcal{F}$. We also denote $C_{b,\mathrm{Lip}}(\mathbb{R}^{n})$ as the linear space of bounded Lipschitz continuous functions *φ* satisfying

$$ \bigl\vert \varphi (\boldsymbol{x})-\varphi (\boldsymbol{y}) \bigr\vert \leq C \vert \boldsymbol{x}-\boldsymbol{y} \vert , \quad \forall \boldsymbol{x}, \boldsymbol{y}\in \mathbb{R}^{n}, $$

for some $C>0$.

### Definition 2.1

A functional $\mathbb{E}$: $\mathcal{H} \rightarrow \overline{ \mathbb{R}}$ is said to be a sub-linear expectation if it satisfies: for $\forall X,Y\in \mathcal{H}$,

- Monotonicity: $X\geq Y$ implies $\mathbb{E}[X]\geq \mathbb{E}[Y]$.
- Constant preserving: $\mathbb{E}[c]=c$, $\forall c\in \mathbb{R}$.
- Positive homogeneity: $\mathbb{E}[\lambda X]=\lambda \mathbb{E}[X]$, $\forall \lambda \geq 0$.
- Sub-additivity: $\mathbb{E}[X+Y]\leq \mathbb{E}[X]+\mathbb{E}[Y]$ whenever $\mathbb{E}[X]+\mathbb{E}[Y]$ is well defined.

The triple $(\varOmega, \mathcal{H}, \mathbb{E})$ is called a sub-linear expectation space.

### Remark 2.1

The sub-linear expectation $\mathbb{E}[\cdot ]$ satisfies translation invariance: $\mathbb{E}[X+c]=\mathbb{E}[X]+c$, $\forall c\in \mathbb{R}$.

### Definition 2.2

([3])

A set function $V:\mathcal{F}\rightarrow [0,1]$ is called a capacity if it satisfies

- $V(\emptyset)=0$, $V(\varOmega)=1$.
- $V(A)\leq V(B)$, $A\subset B$, $A,B\in \mathcal{F}$.

### Definition 2.3

For a capacity *V*, a set *A* is a polar set if $V(A)=0$. And we say a property holds “quasi-surely” (q.s.) if it holds outside a polar set.

### Definition 2.4

A sub-linear expectation $\mathbb{E}:\mathcal{H}\rightarrow \overline{ \mathbb{R}}$ is said to be continuous if it satisfies:

1. continuity from below: $X_{n}\uparrow X$ implies $\mathbb{E}[X _{n}]\uparrow \mathbb{E}[X]$, where $0\leq X_{n}$, $X\in \mathcal{H}$.
2. continuity from above: $X_{n}\downarrow X$ implies $\mathbb{E}[X _{n}]\downarrow \mathbb{E}[X]$, where $0\leq X_{n}$, $X\in \mathcal{H}$.

A capacity $V:\mathcal{F}\rightarrow [0,1]$ is said to be continuous if it satisfies:

1. continuity from below: $A_{n}\uparrow A$ implies $V(A_{n})\uparrow V(A)$, where $A_{n},A\in \mathcal{F}$.
2. continuity from above: $A_{n}\downarrow A$ implies $V(A_{n}) \downarrow V(A)$, where $A_{n},A\in \mathcal{F}$.

The conjugate expectation $\mathcal{E}$ of sub-linear expectation $\mathbb{E}$ is defined by

$$ \mathcal{E}[X]:=-\mathbb{E}[-X], \quad \forall X\in \mathcal{H}. $$

Obviously, for all $X\in \mathcal{H}$, $\mathcal{E}[X]\leq \mathbb{E}[X]$. A pair of capacities can be induced as follows: $\mathbb{V}(A):=\mathbb{E}[I_{A}]$, $v(A):=\mathcal{E}[I _{A}]=1-\mathbb{V}(A^{c})$, $\forall A\in \mathcal{F}$.

### Definition 2.5

([15–18])

(Independence) $\boldsymbol{Y}=(Y_{1},\ldots,Y_{n})$ ($Y_{i} \in \mathcal{H}$) is said to be independent of $\boldsymbol{X}=(X_{1},\ldots,X _{m})$ ($X_{i}\in \mathcal{H}$) if, for each test function $\varphi \in C_{l,\mathrm{Lip}}(\mathbb{R}^{m} \times \mathbb{R}^{n})$,

$$ \mathbb{E}\bigl[\varphi (\boldsymbol{X},\boldsymbol{Y})\bigr]=\mathbb{E}\bigl[ \mathbb{E}\bigl[\varphi ({\boldsymbol{x},\boldsymbol{Y}})\bigr]|_{ \boldsymbol{x}=\boldsymbol{X}} \bigr], $$

whenever the sub-linear expectations are finite.

$\{X_{n}\}_{n=1}^{\infty }$ is said to be a sequence of independent random variables if $X_{n+1}$ is independent of $(X_{1},\ldots,X_{n})$ for each $n\geq 1$.

Let ***X*** be an *n*-dimensional random variable on a sub-linear expectation space $(\varOmega, \mathcal{H}, \mathbb{E})$. We define a functional on $C_{l,\mathrm{Lip}}(\mathbb{R}^{n})$ such that

$$ \mathbb{F}_{\boldsymbol{X}}[\varphi ]:=\mathbb{E}\bigl[\varphi ( \boldsymbol{X}) \bigr], \quad \varphi \in C_{l,\mathrm{Lip}}\bigl(\mathbb{R}^{n}\bigr) \rightarrow \mathbb{R}. $$

Then $\mathbb{F}_{\boldsymbol{X}}[\cdot ]$ can be regarded as the distribution of ***X*** under $\mathbb{E}$ and it characterizes the uncertainty of the distribution of ***X***.

### Definition 2.6

([15–18])

(Identical distribution) Two *n*-dimensional random variables $\boldsymbol{X}_{1}$, $\boldsymbol{X}_{2}$ on respective sub-linear expectation spaces $(\varOmega_{1}, \mathcal{H}_{1}, \mathbb{E}_{1})$ and $(\varOmega_{2}, \mathcal{H}_{2}, \mathbb{E}_{2})$ are called identically distributed, denoted by $\boldsymbol{X}_{1}\overset{d}{=} \boldsymbol{X}_{2}$, if

$$ \mathbb{E}_{1}\bigl[\varphi (\boldsymbol{X}_{1})\bigr]= \mathbb{E}_{2}\bigl[\varphi ( \boldsymbol{X}_{2})\bigr], \quad \forall \varphi \in C_{l,\mathrm{Lip}}\bigl(\mathbb{R}^{n}\bigr), $$

whenever the sub-linear expectations are finite.

### Definition 2.7

([15–18])

A one-dimensional random variable *ξ* on sub-linear expectation $(\varOmega, \mathcal{H}, \mathbb{E})$ is said to be *G*-normal distributed, denoted by $\xi \sim \mathcal{N}(0,[\underline{ \sigma }^{2},\overline{\sigma }^{2}])$, if for any $\varphi \in C_{l,\mathrm{Lip}}( \mathbb{R})$ the following function defined by

$$ u(t,x):=\mathbb{E}\bigl[\varphi (x+\sqrt{t}\xi)\bigr], \quad (t,x)\in [0,\infty) \times \mathbb{R}, $$

is the unique viscosity solution of the following parabolic partial differential equation (PDE) defined on $[0,\infty)\times R$:

$$ \textstyle\begin{cases} \partial_{t} u-G(\partial_{xx}u)=0, \\ u|_{t=0}=\varphi, \end{cases} $$

where $G(a)=\frac{1}{2}a^{+}\overline{\sigma }^{2}-\frac{1}{2}a^{-}\underline{ \sigma }^{2}$, $a\in \mathbb{R}$.

### Remark 2.2

The *G*-normal distributed random variable *ξ* satisfies: $a\xi +b\overline{\xi }\overset{d}{=}\sqrt{a^{2}+b^{2}}\xi$, $\forall a,b\geq 0$, where $\overline{\xi }\overset{d}{=}\xi $ and *ξ̅* is independent of *ξ*. This implies $\mathbb{E}[\xi ]= \mathbb{E}[-\xi ]=0$.

Next we recall the definition of G-expectation. Let $\widetilde{\varOmega }=C[0,\infty)$ be a space of all $\mathbb{R}$-valued continuous paths $(\omega_{t})_{t\geq 0}$ with $\omega_{0}=0$, equipped with distance

$$ \rho \bigl(\omega^{1},\omega^{2}\bigr):=\sum _{i=1}^{\infty }2^{-i}\Bigl[\Bigl( \max _{t\in [0,i]} \bigl\vert \omega_{t}^{1}- \omega_{t}^{2} \bigr\vert \Bigr)\wedge 1\Bigr]. $$

Denote $W_{t}(\omega):=\omega_{t}$ for each $\omega \in \widetilde{\varOmega }$ and

$$ L_{ip}(\widetilde{\varOmega }):=\bigl\{ \varphi (W_{t_{1}}, \ldots, W_{t_{k}}):k \in \mathbb{N}, t_{1},\ldots, t_{k} \in [0,\infty),\varphi \in C_{b,\mathrm{Lip}}\bigl( \mathbb{R}^{k}\bigr) \bigr\} . $$

For each given monotonic and sub-linear function $G(a)=\frac{1}{2}a ^{+}\overline{\sigma }^{2}-\frac{1}{2}a^{-}\underline{\sigma }^{2}$, $0\leq \underline{\sigma }\leq \overline{\sigma }<\infty $, $a\in \mathbb{R}$, let the canonical process $(W_{t})_{t\geq 0}$ be G-Brownian motion on a G-expectation space $(\widetilde{\varOmega },L _{ip}(\widetilde{\varOmega }),\widetilde{\mathbb{E}})$. That is,

$$ \widetilde{\mathbb{E}}\bigl[\varphi (W_{t_{1}},\ldots,W_{t_{n-1}},W_{t_{n}}-W _{t_{n-1}})\bigr]=\widetilde{\mathbb{E}}\bigl[\psi (W_{t_{1}}, \ldots,W_{t_{n-1}})\bigr], $$

where $\psi (x_{1},\ldots,x_{n-1})=\widetilde{\mathbb{E}}[\varphi (x_{1},\ldots,x _{n-1},\sqrt{t_{n}-t_{n-1}}W_{1})]$, $W_{1}\sim \mathcal{N}(\{0\};[\underline{ \sigma }^{2},\overline{\sigma }^{2}])$.

For each $p\geq 1$, we denote $L_{G}^{p}(\widetilde{\varOmega })$ as the completion of $L_{ip}(\widetilde{\varOmega })$ under the norm $\|X\|_{L _{G}^{p}}:=(\widetilde{\mathbb{E}}[|X|^{p}])^{1/p}$. Then the G-expectation $\widetilde{\mathbb{E}}$ can be continuously extended to $(\widetilde{\varOmega },L_{G}^{p}(\widetilde{\varOmega }))$. We still denote the extended G-expectation space by $(\widetilde{\varOmega },L_{G}^{p}( \widetilde{\varOmega }),\widetilde{\mathbb{E}})$.

### Proposition 2.1

([4, 11])

*There exists a weakly compact set of probability measures* $\mathcal{P}$ *on* $(\widetilde{\varOmega },\mathcal{B}( \widetilde{\varOmega }))$ *such that*

$$ \widetilde{\mathbb{E}}[X]=\sup_{P\in \mathcal{P}}E_{P}[X] \quad \textit{for any } X\in L_{G}^{1}(\widetilde{\varOmega }), $$

*where* $\mathcal{B}(\widetilde{\varOmega })$ *denotes the Borel* *σ*-*algebra of* *Ω̃*. *We say that* $\mathcal{P}$ *represents* $\widetilde{\mathbb{E}}$.

Given a G-expectation space $(\widetilde{\varOmega },L_{G}^{1}( \widetilde{\varOmega }),\widetilde{\mathbb{E}})$, we can define a pair of capacities:

$$ \widetilde{\mathbb{V}}(A):=\sup_{P\in \mathcal{P}}P(A)= \widetilde{ \mathbb{E}}[I_{A}], \qquad \widetilde{v}(A):=\inf_{P\in \mathcal{P}}P(A)=1- \widetilde{\mathbb{V}}\bigl(A^{c}\bigr), \quad A\in \mathcal{B}( \widetilde{\varOmega }). $$

Obviously, by Proposition 2.1, $\widetilde{\mathbb{E}}[\cdot ]$ and $\widetilde{\mathbb{V}}(\cdot)$ are continuous from below.

### Definition 2.8

A sub-linear expectation $\mathbb{E}$ is said to be regular if, for each sequence $\{X_{n}\}_{n=1}^{\infty }$ satisfying $X_{n}\downarrow 0$, we have $\mathbb{E}[X_{n}]\downarrow 0$.

### Lemma 2.1

([4, 11])

1. *For any closed sets* $F_{n}\downarrow F$, *it holds that* $\widetilde{\mathbb{V}}(F_{n})\downarrow \widetilde{\mathbb{V}}(F)$.
2. *G*-*expectation* $\widetilde{\mathbb{E}}[\cdot ]$ *is regular*.

Hu et al. [11] indicated that *G*-Brownian motion does not converge to any single point in probability under capacity $\widetilde{\mathbb{V}}$ as follows.

### Lemma 2.2

*Given a G*-*expectation space* $(\widetilde{\varOmega },L_{G}^{2}( \widetilde{\varOmega }),\widetilde{\mathbb{E}})$, *for any fixed* $a\in \mathbb{R}$, *it holds that*

$$ \lim_{\varepsilon \searrow 0}\sup_{a\in \mathbb{R}} \widetilde{\mathbb{V}} \bigl( \vert W_{t}-a \vert \leq \varepsilon\bigr)=0, \quad \forall t>0. $$

*In particular*, *the above equation holds for G*-*normal distribution* $W_{1}$.

### Lemma 2.3

([8])

$\mathbb{E}[|X|]<\infty $ *implies* $|X|<\infty $ *q*.*s*., *i*.*e*., $\mathbb{V}(|X|=\infty)=0$.

The following Rosenthal’s inequality under sub-linear expectation was obtained by Zhang [24].

### Proposition 2.2

*Assume that* $\{X_{n}\}_{n=1}^{\infty }$ *is a sequence of independent random variables*. *Denote* $S_{n}:=\sum_{i=1}^{n} X_{i}$. *Then*, *for any* $p\geq 2$, *we have*

2.1 $$\begin{aligned}& \mathbb{E}\Bigl[\max_{i\leq n} \vert S_{i} \vert ^{p}\Bigr] \\& \quad \leq C_{p}\Biggl\{ \sum _{i=1}^{n} \mathbb{E}\bigl[ \vert X_{i} \vert ^{p}\bigr]+\Biggl(\sum_{i=1}^{n} \mathbb{E}\bigl[ \vert X_{i} \vert ^{2}\bigr] \Biggr)^{ \frac{p}{2}}+\Biggl(\sum_{i=1}^{n} \bigl[\bigl(\mathbb{E}[X_{i}]\bigr)^{+}+\bigl(\mathcal{E}[X _{i}]\bigr)^{-}\bigr]\Biggr)^{p}\Biggr\} , \end{aligned}$$

*where* $C_{p}$ *is a positive constant depending on* *p*.

### Lemma 2.4

*Assume that* $\mathbb{E}$ *is continuous from below and* $\lim_{n\rightarrow \infty }X_{n}=X$. *Then*

$$ \mathbb{E}[X]\leq \liminf_{n\rightarrow \infty }\mathbb{E}[X_{n}]. $$

*If we further assume that* $\mathbb{E}$ *is continuous*, *then*

$$ \mathbb{E}[X]= \lim_{n\rightarrow \infty }\mathbb{E}[X_{n}]. $$

### Proof

Since $\inf_{i\geq n}X_{i}$ is non-decreasing in *n*, we have

$$\begin{aligned} \mathbb{E}[X]&= \mathbb{E}\Bigl[\liminf_{n\rightarrow \infty }X_{n} \Bigr]= \mathbb{E}\Bigl[\lim_{n\rightarrow \infty }\inf_{i\geq n}X_{i} \Bigr]= \lim_{n\rightarrow \infty }\mathbb{E}\Bigl[\inf_{i\geq n}X_{i} \Bigr] \\ &= \liminf_{n\rightarrow \infty }\mathbb{E}\Bigl[\inf_{i\geq n}X_{i} \Bigr] \leq \liminf_{n\rightarrow \infty }\mathbb{E}[X_{n}]. \end{aligned}$$

If $\mathbb{E}$ is continuous, by noting that $\sup_{i\geq n}X_{i}$ is non-increasing in *n*, we have

$$\begin{aligned} \mathbb{E}[X]&= \mathbb{E}\Bigl[\limsup_{n\rightarrow \infty }X_{n} \Bigr]= \mathbb{E}\Bigl[\lim_{n\rightarrow \infty }\sup_{i\geq n}X_{i} \Bigr]= \lim_{n\rightarrow \infty }\mathbb{E}\Bigl[\sup_{i\geq n}X_{i} \Bigr]= \limsup_{n\rightarrow \infty }\mathbb{E}\Bigl[\sup_{i\geq n}X_{i} \Bigr] \\ &\geq \limsup_{n\rightarrow \infty }\mathbb{E}[X_{n}]\geq \liminf _{n\rightarrow \infty }\mathbb{E}[X_{n}]\geq \mathbb{E}[X]. \end{aligned}$$

Thus $\lim_{n\rightarrow \infty }\mathbb{E}[X_{n}]=\mathbb{E}[X]$. □

### Lemma 2.5

*Assume that* $\mathbb{E}$ *is continuous from below and regular*. *Let* $\{X_{n}\}_{n=1}^{\infty }$ *be a sequence of independent random variables with* $\mathbb{E}[X_{n}]=\mathcal{E}[X_{n}]=0$ *for any* $n\geq 1$ *and* $\sum_{i=1}^{\infty }\mathbb{E}[X_{i}^{2}]<\infty $. *Then* $S:=\sum_{i=1}^{\infty }X_{i}$ *convergence q*.*s*. *under capacity* $\mathbb{V}$ *and*, *for any* $p\geq 2$, *we have*

2.2 $$ \mathbb{E}\bigl[ \vert S \vert ^{p}\bigr]\leq C_{p} \Biggl\{ \sum_{i=1}^{\infty }\mathbb{E}\bigl[ \vert X_{i} \vert ^{p}\bigr]+\Biggl( \sum _{i=1}^{\infty }\mathbb{E}\bigl[ \vert X_{i} \vert ^{2}\bigr]\Biggr)^{\frac{p}{2}}\Biggr\} . $$

### Proof

One can refer to Zhang and Lin [25] for the proof of the convergence of *S*. Now we prove (2.2). By $\mathbb{E}[X_{n}]=\mathcal{E}[X_{n}]=0$, taking $\limsup_{n\rightarrow \infty }$ on both sides of (2.1), we have

$$ \limsup_{n\rightarrow \infty }\mathbb{E}\Bigl[\max_{i\leq n} \vert S_{i} \vert ^{p}\Bigr] \leq C_{p}\Biggl\{ \sum_{i=1}^{\infty }\mathbb{E}\bigl[ \vert X_{i} \vert ^{p}\bigr]+\Biggl(\sum _{i=1} ^{\infty }\mathbb{E}\bigl[ \vert X_{i} \vert ^{2}\bigr]\Biggr)^{\frac{p}{2}}\Biggr\} . $$

On the other hand,

$$ \mathbb{E}\Bigl[\max_{i\leq n} \vert S_{i} \vert ^{p}\Bigr]\geq \mathbb{E}\bigl[ \vert S_{n} \vert ^{p}\bigr]. $$

Note that $\lim_{n\rightarrow \infty }S_{n}=S$. By Lemma 2.4 we have

$$ \liminf_{n\rightarrow \infty }\mathbb{E}\Bigl[\max_{i\leq n} \vert S_{i} \vert ^{p}\Bigr] \geq \liminf _{n\rightarrow \infty }\mathbb{E}\bigl[ \vert S_{n} \vert ^{p}\bigr]\geq \mathbb{E}\bigl[ \vert S \vert ^{p} \bigr]. $$

Combining the above inequalities, we have

$$ \mathbb{E}\bigl[ \vert S \vert ^{p}\bigr]\leq C_{p} \Biggl\{ \sum_{i=1}^{\infty }\mathbb{E}\bigl[ \vert X_{i} \vert ^{p}\bigr]+\Biggl( \sum _{i=1}^{\infty }\mathbb{E}\bigl[ \vert X_{i} \vert ^{2}\bigr]\Biggr)^{\frac{p}{2}}\Biggr\} . $$

 □

Throughout the rest of this paper, let $\{X_{n}\}_{n=1}^{\infty }$ be a sequence of independent random variables on a sub-linear expectation space $(\varOmega,\mathcal{H},\mathbb{E})$ with $\mathbb{E}[X_{n}]= \mathcal{E}[X_{n}]=0$, $\mathbb{E}[X_{n}^{2}]=\overline{\sigma }_{n} ^{2}$, $\mathcal{E}[X_{n}^{2}]=\underline{\sigma }_{n}^{2}$, $0<\underline{\sigma }_{n}\leq \overline{\sigma }_{n}<\infty $. Denote $S_{n}:=\sum_{i=1}^{n} X_{i}$, $\overline{B}_{n}^{2}:=\sum_{i=1}^{n} \overline{ \sigma }_{i}^{2}$, and $\underline{B}_{n}^{2}:=\sum_{i=1}^{n} \underline{\sigma}_{i}^{2}$. The symbol *C* presents an arbitrary positive constant and may take different values in different positions.

Zhang [23] obtained the following CLT for sub-linear expectation under the Lindeberg condition as a corollary of the martingale CLT for sub-linear expectation.

### Theorem 2.1

*Let* *ξ* *be* *G*-*normal distributed on a G*-*expectation space* $(\widetilde{\varOmega },L_{G}^{2}(\widetilde{\varOmega }), \widetilde{\mathbb{E}})$ *with* $\xi \sim \mathcal{N}(\{0\};[\underline{ \sigma }^{2},1])$, $0<\underline{\sigma }\leq 1$. *Assume that*

1. 2.3 $$ \lim_{n\rightarrow \infty }\frac{1}{\overline{B}_{n}^{2}}\sum_{i=1} ^{n} \bigl\vert \underline{\sigma }^{2}\cdot \overline{ \sigma }_{i}^{2}-\underline{ \sigma }_{i}^{2} \bigr\vert =0. $$
2. *For any* $\varepsilon >0$,
  2.4 $$ \lim_{n\rightarrow \infty }\frac{1}{\overline{B}_{n}^{2}}\sum_{i=1} ^{n} \mathbb{E}\bigl[ \vert X_{i} \vert ^{2}I \bigl( \vert X_{i} \vert >\varepsilon \overline{B}_{n} \bigr)\bigr]=0. $$

*Then*, *for any* $\varphi \in C_{b,\mathrm{Lip}}(\mathbb{R})$,

2.5 $$ \lim_{n\rightarrow \infty }\mathbb{E}\biggl[\varphi \biggl(\frac{S_{n}}{ \overline{B}_{n}} \biggr)\biggr]=\widetilde{\mathbb{E}}\bigl[\varphi (\xi)\bigr]. $$

## The bound on the distance between the normalized sum distribution and G-normal distribution

The following theorem gives a bound on the distance between the normalized sum distribution $\mathbb{E}[\varphi (\frac{S_{n}}{ \overline{B}_{n}})]$ and G-normal distribution $\widetilde{\mathbb{E}}[\varphi (\xi)]$ where $\xi \sim \mathcal{N}( \{0\};[\underline{\sigma }^{2},1])$.

### Theorem 3.1

*Let* *ξ* *be* *G*-*normal distributed on a G*-*expectation space* $(\widetilde{\varOmega },L_{G}^{2}(\widetilde{\varOmega }), \widetilde{\mathbb{E}})$ *with* $\xi \sim \mathcal{N}(\{0\};[\underline{ \sigma }^{2},1])$, $0<\underline{\sigma }\leq 1$.

*Then*, *for any fixed* $\varphi \in C_{b,\mathrm{Lip}}(\mathbb{R})$ *and any* $h>0$, $0<\varepsilon <1$, *there exist some* $0<\alpha < 1$, $C>0$, *and* $C_{h}>0$ (*a positive constant depending on* *h*) *such that*

3.1 $$\begin{aligned} & \biggl\vert \mathbb{E}\biggl[\varphi \biggl(\frac{S_{n}}{\overline{B}_{n}}\biggr)\biggr]- \widetilde{\mathbb{E}}\bigl[\varphi (\xi)\bigr] \biggr\vert \\ &\quad \leq \frac{C_{h}}{\overline{B}_{n}^{2}}\sum_{i=1}^{n} \bigl\vert \underline{ \sigma }^{2}\cdot \overline{\sigma }_{i}^{2}-\underline{\sigma }_{i} ^{2} \bigr\vert +\frac{C_{h}}{\overline{B}_{n}^{2}}\sum_{i=1}^{n} \mathbb{E}\bigl[ \vert X _{i} \vert ^{2}I\bigl( \vert X_{i} \vert >\varepsilon \overline{B}_{n}\bigr)\bigr] \\ &\qquad {}+C_{h}\Biggl(\frac{1}{\overline{B}_{n}^{2}}\sum _{i=1}^{n} \mathbb{E}\bigl[ \vert X _{i} \vert ^{2}I\bigl( \vert X_{i} \vert > \varepsilon \overline{B}_{n}\bigr)\bigr]\Biggr)^{1+ \frac{\alpha }{2}}+C_{h} \varepsilon^{\alpha }+C(\sqrt{h}+ \sqrt{1+h}-1). \end{aligned}$$

### Remark 3.1

By Theorem 3.1 we can derive Theorem 2.1. If (2.3) and (2.4) hold, taking $n\rightarrow \infty $, $\varepsilon \rightarrow 0$, and $h\rightarrow 0$ in turn on both sides of (3.1), we can get (2.5).

### Proof

For any fixed $\varphi \in C_{b,\mathrm{Lip}}(\mathbb{R})$ and any $h>0$, let $V(t,x)=\widetilde{\mathbb{E}}[\varphi (x+\sqrt{1+h-t}\xi)]$. By Definition 2.7, we have that *V* is the unique viscosity solution of the following parabolic PDE:

3.2 $$ \textstyle\begin{cases} \partial_{t} V+\frac{1}{2}(\partial_{xx}V)^{+}-\frac{1}{2}(\partial _{xx}V)^{-}\underline{\sigma }^{2}=0, \\ V|_{t=1+h}=\varphi. \end{cases} $$

Let $X_{i}^{(n)}=(-\overline{B}_{n})\vee (X_{i} \wedge \overline{B} _{n})$, $S_{i}^{(n)}=\frac{1}{\overline{B}_{n}}\sum_{j=1}^{i} X_{j} ^{(n)}$, $S_{0}^{(n)}=0$, $\delta_{i}^{(n)}=\frac{1}{\overline{B}_{n} ^{2}}\sum_{j=1}^{i} \overline{\sigma }_{j}^{2}$, $\delta_{0}^{(n)}=0$ for each $i=1,2,\ldots,n$. Then we have

$$\begin{aligned} &\biggl\vert \mathbb{E}\biggl[\varphi \biggl(\frac{S_{n}}{\overline{B}_{n}}\biggr)\biggr]- \widetilde{\mathbb{E}}\bigl[\varphi (\xi)\bigr] \biggr\vert \\ &\quad \leq \biggl\vert \mathbb{E}\biggl[\varphi \biggl(\frac{S _{n}}{\overline{B}_{n}}\biggr)\biggr]- \mathbb{E}\bigl[\varphi \bigl(S_{n}^{(n)}\bigr)\bigr] \biggr\vert + \bigl\vert \mathbb{E}\bigl[\varphi \bigl(S_{n}^{(n)} \bigr)\bigr]-\widetilde{\mathbb{E}}\bigl[\varphi ( \xi)\bigr] \bigr\vert \\ &\quad = \biggl\vert \mathbb{E}\biggl[\varphi \biggl(\frac{S_{n}}{\overline{B}_{n}}\biggr) \biggr]-\mathbb{E}\bigl[ \varphi \bigl(S_{n}^{(n)}\bigr)\bigr] \biggr\vert + \bigl\vert \mathbb{E}\bigl[V\bigl(1+h,S_{n}^{(n)} \bigr)\bigr]-V(h,0) \bigr\vert \\ &\quad \leq \biggl\vert \mathbb{E}\biggl[\varphi \biggl(\frac{S_{n}}{\overline{B}_{n}}\biggr) \biggr]- \mathbb{E}\bigl[\varphi \bigl(S_{n}^{(n)}\bigr)\bigr] \biggr\vert + \bigl\vert \mathbb{E}\bigl[V\bigl(1+h,S_{n}^{(n)} \bigr)\bigr]- \mathbb{E}\bigl[V\bigl(1,S_{n}^{(n)}\bigr)\bigr] \bigr\vert \\ &\qquad {}+ \bigl\vert \mathbb{E}\bigl[V\bigl(1,S_{n}^{(n)}\bigr) \bigr]-V(0,0) \bigr\vert + \bigl\vert V(0,0)-V(h,0) \bigr\vert . \end{aligned}$$

Since $\varphi \in C_{b,\mathrm{Lip}}(\mathbb{R})$, for any $0<\varepsilon <1$, it holds that

$$\begin{aligned} & \biggl\vert \mathbb{E}\biggl[\varphi \biggl(\frac{S_{n}}{\overline{B}_{n}}\biggr)\biggr]- \mathbb{E}\bigl[ \varphi \bigl(S_{n}^{(n)}\bigr)\bigr] \biggr\vert \\ &\quad \leq \frac{C}{\overline{B}_{n}}\sum_{i=1} ^{n}\mathbb{E}\bigl[ \bigl\vert X_{i}-X_{i}^{(n)} \bigr\vert \bigr]\leq \frac{C}{\overline{B}_{n}} \sum_{i=1}^{n} \mathbb{E}\bigl[ \vert X_{i} \vert I\bigl( \vert X_{i} \vert >\overline{B}_{n}\bigr)\bigr] \\ &\quad \leq \frac{C}{\overline{B}_{n}^{2}}\sum_{i=1}^{n} \mathbb{E}\bigl[ \vert X _{i} \vert ^{2}I\bigl( \vert X_{i} \vert >\overline{B}_{n}\bigr)\bigr]\leq \frac{C}{\overline{B}_{n} ^{2}}\sum_{i=1}^{n} \mathbb{E} \bigl[ \vert X_{i} \vert ^{2}I\bigl( \vert X_{i} \vert >\varepsilon \overline{B}_{n}\bigr)\bigr], \\ & \bigl\vert \mathbb{E}\bigl[V\bigl(1+h,S_{n}^{(n)}\bigr) \bigr]-\mathbb{E}\bigl[V\bigl(1,S_{n}^{(n)}\bigr)\bigr] \bigr\vert \\ &\quad \leq \sup_{x} \bigl\vert V(1+h,x)-V(1,x) \bigr\vert =\sup_{x} \bigl\vert \varphi (x)- \widetilde{ \mathbb{E}}\bigl[\varphi (x+\sqrt{h}\xi)\bigr] \bigr\vert \\ &\quad \leq \sup_{x} \widetilde{\mathbb{E}}\bigl[ \bigl\vert \varphi (x)-\varphi (x+ \sqrt{h}\xi) \bigr\vert \bigr]\leq C\sqrt{h} \widetilde{\mathbb{E}}\bigl[ \vert \xi \vert \bigr]\leq C \sqrt{h}, \end{aligned}$$

and

$$\begin{aligned} \bigl\vert V(0,0)-V(h,0) \bigr\vert =& \bigl\vert \widetilde{\mathbb{E}} \bigl[\varphi (\sqrt{1+h}\xi)\bigr]- \widetilde{\mathbb{E}}\bigl[\varphi (\xi) \bigr] \bigr\vert \\ \leq& C(\sqrt{1+h}-1) \widetilde{\mathbb{E}}\bigl[ \vert \xi \vert \bigr]\leq C(\sqrt{1+h}-1). \end{aligned}$$

Then

3.3 $$\begin{aligned} \biggl\vert \mathbb{E}\biggl[\varphi \biggl(\frac{S_{n}}{\overline{B}_{n}}\biggr)\biggr]- \widetilde{\mathbb{E}}\bigl[\varphi (\xi)\bigr] \biggr\vert \leq{} & \bigl\vert \mathbb{E}\bigl[V\bigl(1,S_{n} ^{(n)}\bigr) \bigr]-V(0,0) \bigr\vert +\frac{C}{\overline{B}_{n}^{2}}\sum_{i=1}^{n} \mathbb{E}\bigl[ \vert X_{i} \vert ^{2}I\bigl( \vert X_{i} \vert >\varepsilon \overline{B}_{n}\bigr)\bigr] \\ &{}+C(\sqrt{h}+\sqrt{1+h}-1). \end{aligned}$$

So it is sufficient to get the bound of $|\mathbb{E}[V(1,S_{n}^{(n)})]-V(0,0)|$.

$$\begin{aligned} &V\bigl(1,S_{n}^{(n)}\bigr)-V(0,0) \\ &\quad =\sum _{i=0}^{n-1} \bigl[V\bigl(\delta_{i+1}^{(n)},S_{i+1} ^{(n)}\bigr)-V\bigl(\delta_{i}^{(n)},S_{i}^{(n)} \bigr)\bigr] \\ &\quad =\sum_{i=0}^{n-1} \bigl[\bigl(V\bigl( \delta_{i+1}^{(n)},S_{i+1}^{(n)}\bigr)-V\bigl( \delta _{i}^{(n)},S_{i+1}^{(n)}\bigr)\bigr)+ \bigl(V\bigl(\delta_{i}^{(n)},S_{i+1}^{(n)} \bigr)-V\bigl(\delta _{i}^{(n)},S_{i}^{(n)} \bigr)\bigr)\bigr] \\ &\quad :=\sum_{i=0}^{n-1}\bigl(I^{(n)}_{i}+J_{i}^{(n)} \bigr), \end{aligned}$$

where $I_{i}^{(n)}$ and $J_{i}^{(n)}$ are obtained by Taylor expansion:

$$\begin{aligned} &I_{i}^{(n)}= \frac{\overline{\sigma }_{i+1}^{2}}{\overline{B}^{2}_{n}}\partial_{t} V \bigl(\delta_{i}^{(n)},S_{i}^{(n)}\bigr)+ \frac{1}{2\overline{B}^{2}_{n}} \partial_{xx}V\bigl(\delta_{i}^{(n)},S_{i}^{(n)} \bigr) \bigl\vert X_{i+1}^{(n)} \bigr\vert ^{2}+ \frac{1}{ \overline{B}_{n}}\partial_{x} V\bigl(\delta_{i}^{(n)},S_{i}^{(n)} \bigr)X_{i+1} ^{(n)} \\ &\hphantom{I_{i}^{(n)}}= \biggl(\frac{\overline{\sigma }_{i+1}^{2}}{\overline{B}^{2}_{n}} \partial_{t} V\bigl( \delta_{i}^{(n)},S_{i}^{(n)}\bigr)+ \frac{1}{2\overline{B} ^{2}_{n}}\partial_{xx}V\bigl(\delta_{i}^{(n)},S_{i}^{(n)} \bigr)X_{i+1}^{2}+\frac{1}{ \overline{B}_{n}}\partial_{x} V \bigl(\delta_{i}^{(n)},S_{i}^{(n)} \bigr)X_{i+1} \biggr) \\ &\hphantom{I_{i}^{(n)}=}{}+ \biggl(\frac{1}{2\overline{B}^{2}_{n}}\partial_{xx}V\bigl( \delta_{i}^{(n)},S _{i}^{(n)}\bigr) \bigl( \bigl\vert X_{i+1}^{(n)} \bigr\vert ^{2}-X_{i+1}^{2} \bigr)+\frac{1}{\overline{B} _{n}}\partial_{x} V\bigl(\delta_{i}^{(n)},S_{i}^{(n)} \bigr) \bigl(X_{i+1}^{(n)}-X_{i+1}\bigr) \biggr) \\ &\hphantom{I_{i}^{(n)}}:= I_{1,i}^{(n)}+I_{2,i}^{(n)}. \\ &J_{i}^{(n)} = \frac{\overline{\sigma }_{i+1}^{2}}{\overline{B}^{2}_{n}} \bigl[\partial _{t} V\bigl(\delta_{i}^{(n)},S_{i+1}^{(n)} \bigr)-\partial_{t} V\bigl(\delta_{i}^{(n)},S _{i}^{(n)}\bigr)\bigr] \\ &\hphantom{J_{i}^{(n)}=}{}+\frac{\overline{\sigma }_{i+1}^{2}}{\overline{B}^{2}_{n}} \int_{0} ^{1} \biggl[\partial_{t} V \biggl(\delta_{i}^{(n)}+\beta \cdot \frac{\overline{ \sigma }_{i+1}^{2}}{\overline{B}_{n}^{2}},S_{i+1}^{(n)} \biggr)-\partial_{t} V\bigl(\delta_{i}^{(n)},S_{i+1}^{(n)} \bigr)\biggr]\,d\beta \\ &\hphantom{J_{i}^{(n)}=}{}+ \int_{0}^{1} \int_{0}^{1} \biggl[\partial_{xx}V\biggl( \delta_{i}^{(n)},S_{i} ^{(n)}+\gamma \beta \frac{X_{i+1}^{(n)}}{\overline{B}_{n}}\biggr)-\partial _{xx}V\bigl(\delta_{i}^{(n)},S_{i}^{(n)} \bigr)\biggr] \frac{ \vert X_{i+1}^{(n)} \vert ^{2}}{ \overline{B}_{n}^{2}}\gamma \,d\beta \,d\gamma. \end{aligned}$$

Since $X_{i+1}$ is independent of $S_{i}^{(n)}$, we have

$$\begin{aligned} \mathbb{E}\bigl[\partial_{x} V\bigl(\delta_{i}^{(n)},S_{i}^{(n)} \bigr)X_{i+1}\bigr] &= \mathbb{E}\bigl[\mathbb{E}[r\cdot X_{i+1}]|_{r=\partial_{x} V(\delta_{i} ^{(n)},S_{i}^{(n)})}\bigr] \\ &=\mathbb{E}\bigl[\bigl(\partial_{x} V\bigl(\delta_{i}^{(n)},S_{i}^{(n)} \bigr)\bigr)^{+} \mathbb{E}[X_{i+1}]-\bigl( \partial_{x} V\bigl(\delta_{i}^{(n)},S_{i}^{(n)} \bigr)\bigr)^{-} \mathcal{E}[X_{i+1}]\bigr] \\ &=0. \end{aligned}$$

Similarly, we can also have $\mathbb{E}[-\partial_{x} V(\delta_{i} ^{(n)},S_{i}^{(n)})X_{i+1}]=0$. It follows that

$$\begin{aligned} \mathbb{E}\bigl[I_{1,i}^{(n)}\bigr] ={}&\mathbb{E}\biggl[ \frac{\overline{\sigma }_{i+1} ^{2}}{\overline{B}^{2}_{n}}\partial_{t} V\bigl(\delta_{i}^{(n)},S_{i}^{(n)} \bigr)+\frac{1}{2 \overline{B}^{2}_{n}}\partial_{xx}V\bigl(\delta_{i}^{(n)},S_{i}^{(n)} \bigr)X _{i+1}^{2}\biggr] \\ ={}&\frac{1}{\overline{B}^{2}_{n}}\mathbb{E}\biggl[\mathbb{E}\biggl[\overline{ \sigma }_{i+1}^{2} p+\frac{1}{2} qX_{i+1}^{2} \biggr]\bigg|_{p=\partial_{t} V( \delta_{i}^{(n)},S_{i}^{(n)}),q=\partial_{xx}V(\delta_{i}^{(n)},S_{i} ^{(n)})}\biggr] \\ ={}&\frac{1}{\overline{B}^{2}_{n}}\mathbb{E}\biggl[\overline{\sigma }_{i+1} ^{2} p+\frac{1}{2} q^{+}\mathbb{E}\bigl[X_{i+1}^{2} \bigr]-\frac{1}{2} q^{-} \mathcal{E}\bigl[X_{i+1}^{2} \bigr]\bigg|_{p=\partial_{t} V(\delta_{i}^{(n)},S_{i} ^{(n)}),q=\partial_{xx}V(\delta_{i}^{(n)},S_{i}^{(n)})}\biggr] \\ ={}&\frac{1}{\overline{B}^{2}_{n}}\mathbb{E}\biggl[ \partial_{t} V\bigl(\delta _{i}^{(n)},S_{i}^{(n)}\bigr)\overline{ \sigma }_{i+1}^{2}+\frac{1}{2} \bigl( \partial_{xx}V\bigl(\delta_{i}^{(n)},S_{i}^{(n)} \bigr)\bigr)^{+}\overline{\sigma } _{i+1}^{2} \\ &{}- \frac{1}{2} \bigl(\partial_{xx}V\bigl(\delta_{i}^{(n)},S_{i}^{(n)} \bigr)\bigr)^{-}\underline{ \sigma }_{i+1}^{2}\biggr] \\ \leq{}& \frac{\overline{\sigma }_{i+1}^{2}}{\overline{B}^{2}_{n}} \mathbb{E}\biggl[\partial_{t} V\bigl( \delta_{i}^{(n)},S_{i}^{(n)}\bigr)+ \frac{1}{2} \bigl( \partial_{xx}V\bigl(\delta_{i}^{(n)},S_{i}^{(n)} \bigr)\bigr)^{+}-\frac{1}{2} \bigl( \partial_{xx}V\bigl( \delta_{i}^{(n)},S_{i}^{(n)}\bigr) \bigr)^{-}\underline{\sigma } ^{2}\biggr] \\ &{}+\frac{1}{2\overline{B}^{2}_{n}}\mathbb{E}\bigl[\partial_{xx}V\bigl(\delta _{i}^{(n)},S_{i}^{(n)} \bigr)^{-}\underline{\sigma }^{2}\cdot \overline{ \sigma }_{i+1}^{2}-\partial_{xx}V\bigl( \delta_{i}^{(n)},S_{i}^{(n)} \bigr)^{-}\underline{ \sigma }_{i+1}^{2}\bigr] \\ \leq{}& \frac{1}{2\overline{B}^{2}_{n}} \bigl\vert \underline{\sigma }^{2}\cdot \overline{ \sigma }_{i+1}^{2}-\underline{\sigma }_{i+1}^{2} \bigr\vert \cdot \mathbb{E}\bigl[ \bigl\vert \partial_{xx}V\bigl(\delta_{i}^{(n)},S_{i}^{(n)} \bigr) \bigr\vert \bigr]. \end{aligned}$$

By the interior regularity of *V* (see Peng [18]), it holds that

$$ \Vert V \Vert _{C^{1+\frac{\alpha }{2},2+\alpha }([0,1]\times \mathbb{R})}< \infty \quad \text{for some } \alpha \in (0,1), $$

which implies $\partial_{t} V$, $\partial_{x} V$, and $\partial_{xx}V$ are uniformly $\frac{\alpha }{2}$-Hölder continuous in *t* and *α*-Hölder continuous in *x* on $[0,1]\times \mathbb{R}$. For any $n\geq 1$ and $i\leq n$, it holds that

$$\begin{aligned} \mathbb{E}\bigl[ \bigl\vert \partial_{xx}V\bigl( \delta_{i}^{(n)},S_{i}^{(n)}\bigr) \bigr\vert \bigr]\leq{} & \bigl\vert \partial_{xx}V(0,0) \bigr\vert +C_{h}\bigl( \bigl\vert \delta_{i}^{(n)} \bigr\vert ^{\frac{\alpha }{2}}+ \mathbb{E}\bigl[ \bigl\vert S_{i}^{(n)} \bigr\vert ^{\alpha }\bigr]\bigr) \\ \leq{} & C_{h}\bigl(1+\bigl(\mathbb{E}\bigl[ \bigl\vert S_{i}^{(n)} \bigr\vert ^{2}\bigr] \bigr)^{\frac{\alpha }{2}}\bigr). \end{aligned}$$

By Proposition 2.2, we have

$$\begin{aligned} \mathbb{E}\bigl[ \bigl\vert S_{i}^{(n)} \bigr\vert ^{2}\bigr]\leq{} & \frac{C}{\overline{B}_{n}^{2}} \sum _{i=1}^{n}\mathbb{E}\bigl[ \bigl\vert X_{i}^{(n)} \bigr\vert ^{2}\bigr]+ \frac{C}{\overline{B}_{n} ^{2}}\Biggl(\sum_{i=1}^{n}\bigl[ \bigl(\mathbb{E}\bigl[X_{i}^{(n)}\bigr]\bigr)^{+}+ \bigl(\mathbb{E}\bigl[-X_{i} ^{(n)}\bigr]\bigr)^{+} \bigr]\Biggr)^{2} \\ \leq{} &\frac{C}{\overline{B}_{n}^{2}}\sum_{i=1}^{n} \mathbb{E}\bigl[ \vert X_{i} \vert ^{2}\bigr]+ \frac{C}{ \overline{B}_{n}^{2}}\Biggl(\sum_{i=1}^{n} \mathbb{E}\bigl[ \bigl\vert X_{i}-X_{i}^{(n)} \bigr\vert \bigr]\Biggr)^{2} \\ \leq{} &C+C\Biggl(\frac{1}{\overline{B}_{n}}\sum_{i=1}^{n} \mathbb{E}\bigl[ \vert X_{i} \vert I\bigl( \vert X _{i} \vert >\overline{B}_{n}\bigr)\bigr] \Biggr)^{2} \\ \leq{} &C+C\Biggl(\frac{1}{\overline{B}_{n}^{2}}\sum_{i=1}^{n} \mathbb{E}\bigl[ \vert X _{i} \vert ^{2}\bigr] \Biggr)^{2} \\ \leq{} &C. \end{aligned}$$

So we have $\mathbb{E}[|\partial_{xx}V(\delta_{i}^{(n)},S_{i}^{(n)})|] \leq C_{h}$. Similarly $\mathbb{E}[|\partial_{x}V(\delta_{i}^{(n)},S _{i}^{(n)})|]\leq C_{h}$. Then

$$ \mathbb{E}\Biggl[\sum_{i=0}^{n-1}I_{1,i}^{(n)} \Biggr]\leq \sum_{i=0}^{n-1} \mathbb{E} \bigl[I_{1,i}^{(n)}\bigr]\leq \frac{C_{h}}{\overline{B}^{2}_{n}}\sum _{i=1}^{n} \bigl\vert \underline{\sigma }^{2}\cdot \overline{\sigma }_{i}^{2}-\underline{ \sigma }_{i}^{2} \bigr\vert . $$

On the other hand,

$$\begin{aligned} &\mathbb{E}\Biggl[\sum_{i=0}^{n-1}I_{1,i}^{(n)} \Biggr] \\ &\quad = \mathbb{E}\Biggl[\sum_{i=0}^{n-2}I_{1,i}^{(n)}+ \frac{\overline{\sigma } _{n}^{2}}{\overline{B}^{2}_{n}}\partial_{t} V\bigl(\delta_{n-1}^{(n)},S _{n-1}^{(n)}\bigr)+\frac{1}{2\overline{B}^{2}_{n}}\partial_{xx}V \bigl(\delta _{n-1}^{(n)},S_{n-1}^{(n)} \bigr)X_{n}^{2} \\ &\qquad {}+\frac{1}{\overline{B}_{n}} \partial_{x} V \bigl(\delta_{n-1}^{(n)},S_{n-1}^{(n)} \bigr)X_{n}\Biggr] \\ &\quad = \mathbb{E}\Biggl[\sum_{i=0}^{n-2}I_{1,i}^{(n)}+ \frac{\overline{\sigma } _{n}^{2}}{\overline{B}^{2}_{n}}\partial_{t} V\bigl(\delta_{n-1}^{(n)},S _{n-1}^{(n)}\bigr)+\frac{1}{2\overline{B}^{2}_{n}}\partial_{xx}V \bigl(\delta _{n-1}^{(n)},S_{n-1}^{(n)} \bigr)X_{n}^{2}\Biggr] \\ &\quad = \mathbb{E}\Biggl[\sum_{i=0}^{n-2}I_{1,i}^{(n)}+ \frac{\overline{\sigma } _{n}^{2}}{\overline{B}^{2}_{n}}\partial_{t} V\bigl(\delta_{n-1}^{(n)},S _{n-1}^{(n)}\bigr) \\ &\qquad {}+\frac{1}{2\overline{B}^{2}_{n}}\bigl(\partial_{xx}V\bigl( \delta_{n-1}^{(n)},S _{n-1}^{(n)}\bigr) \bigr)^{+}\overline{\sigma }_{n}^{2}- \frac{1}{2\overline{B} ^{2}_{n}}\bigl(\partial_{xx}V\bigl(\delta_{n-1}^{(n)},S_{n-1}^{(n)} \bigr)\bigr)^{-}\underline{ \sigma }_{n}^{2}\Biggr] \\ &\quad = \mathbb{E}\Biggl[\sum_{i=0}^{n-2}I_{1,i}^{(n)}+ \frac{\overline{\sigma } _{n}^{2}}{\overline{B}^{2}_{n}}\biggl(\partial_{t} V\bigl(\delta_{n-1}^{(n)},S _{n-1}^{(n)}\bigr)+\frac{1}{2}\bigl(\partial_{xx}V \bigl(\delta_{n-1}^{(n)},S_{n-1} ^{(n)}\bigr) \bigr)^{+} \\ &\qquad {}-\frac{1}{2}\bigl(\partial_{xx}V\bigl( \delta_{n-1}^{(n)},S_{n-1} ^{(n)}\bigr) \bigr)^{-}\underline{\sigma }^{2}\biggr) \\ &\qquad {}+\frac{1}{2\overline{B}^{2}_{n}}\bigl(\partial_{xx}V\bigl( \delta_{n-1}^{(n)},S _{n-1}^{(n)}\bigr) \bigr)^{-}\underline{\sigma }^{2}\cdot \overline{\sigma }_{n} ^{2}-\frac{1}{2\overline{B}^{2}_{n}}\bigl(\partial_{xx}V \bigl(\delta_{n-1}^{(n)},S _{n-1}^{(n)}\bigr) \bigr)^{-}\underline{\sigma }_{n}^{2}\Biggr] \\ &\quad = \mathbb{E}\Biggl[\sum_{i=0}^{n-2}I_{1,i}^{(n)}+ \frac{1}{2\overline{B} ^{2}_{n}}\bigl(\partial_{xx}V\bigl(\delta_{n-1}^{(n)},S_{n-1}^{(n)} \bigr)\bigr)^{-}\bigl(\underline{ \sigma }^{2}\cdot \overline{ \sigma }_{n}^{2}-\underline{\sigma }_{n} ^{2}\bigr)\Biggr] \\ &\quad \geq \mathbb{E}\Biggl[\sum_{i=0}^{n-2}I_{1,i}^{(n)} \Biggr]-\mathbb{E}\biggl[-\frac{1}{2 \overline{B}^{2}_{n}}\bigl(\partial_{xx}V\bigl( \delta_{n-1}^{(n)},S_{n-1}^{(n)}\bigr) \bigr)^{-}\bigl(\underline{ \sigma }^{2}\cdot \overline{\sigma }_{n}^{2}-\underline{\sigma }_{n} ^{2} \bigr)\biggr] \\ &\quad \geq \mathbb{E}\Biggl[\sum_{i=0}^{n-2}I_{1,i}^{(n)} \Biggr]-\frac{C_{h}}{ \overline{B}^{2}_{n}} \bigl\vert \underline{\sigma }^{2}\cdot \overline{\sigma }_{n}^{2}-\underline{\sigma }_{n}^{2} \bigr\vert \\ &\quad \geq -\frac{C_{h}}{\overline{B}^{2}_{n}}\sum_{i=1}^{n} \bigl\vert \underline{ \sigma }^{2}\cdot \overline{\sigma }_{i}^{2}-\underline{\sigma }_{i} ^{2} \bigr\vert . \end{aligned}$$

So we have

3.4 $$ \Biggl\vert \mathbb{E}\Biggl[\sum_{i=0}^{n-1}I_{1,i}^{(n)} \Biggr] \Biggr\vert \leq \frac{C_{h}}{ \overline{B}^{2}_{n}}\sum_{i=1}^{n} \bigl\vert \underline{\sigma }^{2}\cdot \overline{ \sigma }_{i}^{2}-\underline{\sigma }_{i}^{2} \bigr\vert . $$

Since $|X_{i+1}^{(n)}|^{2}-X_{i+1}^{2}$ is independent of $\partial _{xx}V(\delta_{i}^{(n)},S_{i}^{(n)})$ and $X_{i+1}^{(n)}-X_{i+1}$ is independent of $\partial_{x} V(\delta_{i}^{(n)},S_{i}^{(n)})$, we have

3.5 $$\begin{aligned} \sum_{i=0}^{n-1}\mathbb{E}\bigl[ \bigl\vert I_{2,i}^{(n)} \bigr\vert \bigr]\leq{} & \sum _{i=0}^{n-1} \biggl\{ \frac{1}{2\overline{B}^{2}_{n}}\mathbb{E}\bigl[ \bigl\vert \partial_{xx}V\bigl( \delta_{i}^{(n)},S_{i}^{(n)} \bigr) \bigr\vert \bigr]\mathbb{E}\bigl[ \bigl\vert \bigl\vert X_{i+1}^{(n)} \bigr\vert ^{2}-X_{i+1} ^{2} \bigr\vert \bigr] \\ &{}+\frac{1}{\overline{B}_{n}}\mathbb{E}\bigl[ \bigl\vert \partial_{x} V \bigl(\delta_{i} ^{(n)},S_{i}^{(n)}\bigr) \bigr\vert \bigr]\mathbb{E}\bigl[ \bigl\vert X_{i+1}^{(n)}-X_{i+1} \bigr\vert \bigr] \biggr\} \\ \leq{} & \frac{C_{h}}{\overline{B}_{n}^{2}}\sum_{i=1}^{n} \mathbb{E}\bigl[ \vert X _{i} \vert ^{2}I\bigl( \vert X_{i} \vert >\overline{B}_{n}\bigr)\bigr]+ \frac{C_{h}}{\overline{B}_{n}} \sum_{i=1}^{n}\mathbb{E} \bigl[ \vert X_{i} \vert I\bigl( \vert X_{i} \vert > \overline{B}_{n}\bigr)\bigr] \\ \leq{} &\frac{C_{h}}{\overline{B}_{n}^{2}}\sum_{i=1}^{n} \mathbb{E}\bigl[ \vert X _{i} \vert ^{2}I\bigl( \vert X_{i} \vert >\varepsilon \overline{B}_{n}\bigr)\bigr]. \end{aligned}$$

On the other hand,

$$\begin{aligned}& \biggl\vert \frac{\overline{\sigma }_{i+1}^{2}}{\overline{B}^{2}_{n}} \bigl[\partial _{t} V\bigl( \delta_{i}^{(n)},S_{i+1}^{(n)}\bigr)- \partial_{t} V\bigl(\delta_{i}^{(n)},S _{i}^{(n)}\bigr)\bigr] \biggr\vert \leq C_{h} \cdot \frac{\overline{\sigma }_{i+1}^{2}}{ \overline{B}^{2}_{n}}\cdot \biggl(\frac{ \vert X_{i+1}^{(n)} \vert }{\overline{B}_{n}}\biggr)^{ \alpha }, \\& \biggl\vert \frac{\overline{\sigma }_{i+1}^{2}}{\overline{B}^{2}_{n}} \int_{0} ^{1} \biggl[\partial_{t} V \biggl(\delta_{i}^{(n)}+\beta \cdot \frac{\overline{ \sigma }_{i+1}^{2}}{\overline{B}_{n}^{2}},S_{i+1}^{(n)} \biggr)-\partial_{t} V\bigl(\delta_{i}^{(n)},S_{i+1}^{(n)} \bigr)\biggr]\,d\beta \biggr\vert \leq C_{h}\cdot \frac{\overline{ \sigma }_{i+1}^{2}}{\overline{B}^{2}_{n}} \cdot \biggl(\frac{\overline{ \sigma }_{i+1}^{2}}{\overline{B}^{2}_{n}}\biggr)^{\frac{\alpha }{2}}, \\& \biggl\vert \int_{0}^{1} \int_{0}^{1} \biggl[\partial_{xx}V\biggl( \delta_{i}^{(n)},S_{i}^{(n)}+ \gamma \beta \frac{X_{i+1}^{(n)}}{\overline{B}_{n}}\biggr)-\partial_{xx}V\bigl( \delta_{i}^{(n)},S_{i}^{(n)} \bigr)\biggr] \frac{ \vert X_{i+1}^{(n)} \vert ^{2}}{ \overline{B}_{n}^{2}}\gamma \,d\beta \,d\gamma \biggr\vert \\& \quad \leq C_{h}\cdot \frac{ \vert X _{i+1}^{(n)} \vert ^{2}}{\overline{B}_{n}^{2}}\cdot \biggl( \frac{ \vert X_{i+1}^{(n)} \vert }{ \overline{B}_{n}}\biggr)^{\alpha }. \end{aligned}$$

Then

3.6 $$ \sum_{i=0}^{n-1}\mathbb{E}\bigl[ \bigl\vert J_{i}^{(n)} \bigr\vert \bigr]\leq \frac{C_{h}}{ \overline{B}_{n}^{2+\alpha }} \sum_{i=1}^{n} \overline{\sigma }_{i} ^{2}\mathbb{E}\bigl[ \bigl\vert X_{i}^{(n)} \bigr\vert ^{\alpha }\bigr] +\frac{C_{h}}{\overline{B} _{n}^{2+\alpha }}\sum _{i=1}^{n} \overline{\sigma }_{i}^{2+\alpha }+ \frac{C _{h}}{\overline{B}_{n}^{2+\alpha }}\sum_{i=1}^{n} \mathbb{E} \bigl[ \bigl\vert X_{i} ^{(n)} \bigr\vert ^{2+\alpha } \bigr]. $$

For any $0<\varepsilon <1$, we have

3.7 $$\begin{aligned} &\frac{1}{\overline{B}_{n}^{2+\alpha }}\sum_{i=1}^{n} \mathbb{E}\bigl[ \bigl\vert X _{i}^{(n)} \bigr\vert ^{2+\alpha }\bigr] \\ &\quad =\frac{1}{\overline{B}_{n}^{2+\alpha }}\sum_{i=1}^{n} \mathbb{E}\bigl[ \vert X_{i} \vert ^{2+\alpha }I\bigl( \vert X_{i} \vert \leq \overline{B} _{n}\bigr)+ \overline{B}_{n}^{2+\alpha }I\bigl( \vert X_{i} \vert > \overline{B}_{n}\bigr)\bigr] \\ &\quad \leq \frac{1}{\overline{B}_{n}^{2+\alpha }}\sum_{i=1}^{n} \mathbb{E}\bigl[ \vert X_{i} \vert ^{2+\alpha }I\bigl( \vert X_{i} \vert \leq \varepsilon \overline{B} _{n}\bigr) \bigr]+\frac{1}{\overline{B}_{n}^{2+\alpha }}\sum_{i=1}^{n} \mathbb{E}\bigl[ \vert X_{i} \vert ^{2+\alpha }I\bigl( \varepsilon \overline{B}_{n}< \vert X_{i} \vert \leq \overline{B}_{n}\bigr)\bigr] \\ &\qquad {}+\frac{1}{\overline{B}_{n}^{2+\alpha }}\sum_{i=1}^{n} \mathbb{E}\bigl[ \vert X _{i} \vert ^{2} \overline{B}_{n}^{\alpha }I\bigl( \vert X_{i} \vert > \overline{B}_{n}\bigr)\bigr] \\ &\quad \leq \frac{1}{\overline{B}_{n}^{2+\alpha }}\sum_{i=1}^{n} \varepsilon^{\alpha }\overline{B}_{n}^{\alpha }\mathbb{E} \bigl[ \vert X_{i} \vert ^{2}\bigr]+\frac{1}{ \overline{B}_{n}^{2}} \sum_{i=1}^{n} \mathbb{E}\bigl[ \vert X_{i} \vert ^{2}I\bigl( \vert X_{i} \vert > \varepsilon \overline{B}_{n}\bigr)\bigr] \\ &\qquad {}+\frac{1}{\overline{B}_{n}^{2}}\sum_{i=1} ^{n} \mathbb{E}\bigl[ \vert X_{i} \vert ^{2}I\bigl( \vert X_{i} \vert >\overline{B}_{n}\bigr)\bigr] \\ &\quad \leq \varepsilon^{\alpha }+\frac{C}{\overline{B}_{n}^{2}}\sum _{i=1} ^{n} \mathbb{E}\bigl[ \vert X_{i} \vert ^{2}I\bigl( \vert X_{i} \vert >\varepsilon \overline{B}_{n}\bigr)\bigr]. \end{aligned}$$

By Hölder’s inequality under sub-linear expectation, we have

3.8 $$\begin{aligned} &\frac{1}{\overline{B}_{n}^{2+\alpha }}\sum_{i=1}^{n} \overline{ \sigma }_{i}^{2+\alpha } \\ &\quad =\frac{1}{\overline{B}_{n}^{2+\alpha }}\sum_{i=1}^{n} \bigl(\mathbb{E}\bigl[ \vert X_{i} \vert ^{2}I\bigl( \vert X_{i} \vert \leq \varepsilon \overline{B}_{n} \bigr)+ \vert X_{i} \vert ^{2}I\bigl( \vert X_{i} \vert > \varepsilon \overline{B}_{n}\bigr)\bigr] \bigr)^{1+\frac{ \alpha }{2}} \\ &\quad \leq \frac{C}{\overline{B}_{n}^{2+\alpha }}\sum_{i=1}^{n} \bigl( \mathbb{E}\bigl[ \vert X_{i} \vert ^{2}I\bigl( \vert X_{i} \vert \leq \varepsilon \overline{B}_{n}\bigr) \bigr]\bigr)^{1+\frac{ \alpha }{2}}+\frac{C}{\overline{B}_{n}^{2+\alpha }}\sum_{i=1}^{n} \bigl( \mathbb{E}\bigl[ \vert X_{i} \vert ^{2}I\bigl( \vert X_{i} \vert > \varepsilon \overline{B}_{n}\bigr) \bigr]\bigr)^{1+\frac{ \alpha }{2}} \\ &\quad \leq \frac{C}{\overline{B}_{n}^{2+\alpha }}\sum_{i=1}^{n} \mathbb{E}\bigl[ \vert X_{i} \vert ^{2+\alpha }I\bigl( \vert X_{i} \vert \leq \varepsilon \overline{B} _{n}\bigr) \bigr]+\frac{C}{\overline{B}_{n}^{2+\alpha }}\sum_{i=1}^{n} \bigl( \mathbb{E}\bigl[ \vert X_{i} \vert ^{2}I\bigl( \vert X_{i} \vert > \varepsilon \overline{B}_{n}\bigr) \bigr]\bigr)^{1+\frac{ \alpha }{2}} \\ &\quad \leq C\varepsilon^{\alpha }+C\Biggl(\frac{1}{\overline{B}_{n}^{2}}\sum _{i=1}^{n} \mathbb{E}\bigl[ \vert X_{i} \vert ^{2}I\bigl( \vert X_{i} \vert > \varepsilon \overline{B} _{n}\bigr)\bigr]\Biggr)^{1+\frac{\alpha }{2}}. \end{aligned}$$

3.9 $$\begin{aligned} &\frac{1}{\overline{B}_{n}^{2+\alpha }} \sum_{i=1}^{n} \overline{ \sigma }_{i}^{2}\mathbb{E}\bigl[ \bigl\vert X_{i}^{(n)} \bigr\vert ^{\alpha }\bigr] \\ &\quad \leq \frac{1}{ \overline{B}_{n}^{2+\alpha }}\sum_{i=1}^{n} \overline{\sigma }_{i} ^{2}\mathbb{E}\bigl[ \vert X_{i} \vert ^{\alpha }\bigr]\leq \frac{1}{\overline{B}_{n}^{2+ \alpha }}\sum _{i=1}^{n} \overline{\sigma }_{i}^{2+\alpha } \\ &\quad \leq C\varepsilon^{\alpha }+C\Biggl(\frac{1}{\overline{B}_{n}^{2}}\sum _{i=1}^{n} \mathbb{E}\bigl[ \vert X_{i} \vert ^{2}I\bigl( \vert X_{i} \vert > \varepsilon \overline{B} _{n}\bigr)\bigr]\Biggr)^{1+\frac{\alpha }{2}}. \end{aligned}$$

Combining (3.7), (3.8), and (3.9), we have

3.10 $$\begin{aligned} \sum_{i=0}^{n-1}\mathbb{E}\bigl[ \bigl\vert J_{i}^{(n)} \bigr\vert \bigr] \leq& C_{h} \varepsilon^{ \alpha }+\frac{C_{h}}{\overline{B}_{n}^{2}}\sum_{i=1}^{n} \mathbb{E}\bigl[ \vert X _{i} \vert ^{2}I\bigl( \vert X_{i} \vert >\varepsilon \overline{B}_{n}\bigr)\bigr] \\ &{}+C_{h}\Biggl(\frac{1}{ \overline{B}_{n}^{2}}\sum_{i=1}^{n} \mathbb{E}\bigl[ \vert X_{i} \vert ^{2}I\bigl( \vert X_{i} \vert > \varepsilon \overline{B}_{n}\bigr)\bigr] \Biggr)^{1+\frac{\alpha }{2}}. \end{aligned}$$

By (3.4), (3.5), and (3.10), it holds that

$$\begin{aligned} \bigl\vert \mathbb{E}\bigl[V\bigl(1,S_{n}^{(n)}\bigr) \bigr]-V(0,0) \bigr\vert \leq{} & \Biggl\vert \mathbb{E}\Biggl[\sum _{i=0} ^{n-1}I_{1,i}^{(n)}\Biggr] \Biggr\vert +\sum_{i=0}^{n-1}\mathbb{E} \bigl[ \bigl\vert I_{2,i}^{(n)} \bigr\vert \bigr]+ \sum _{i=0}^{n-1}\mathbb{E}\bigl[ \bigl\vert J_{i}^{(n)} \bigr\vert \bigr] \\ \leq{} &\frac{C_{h}}{\overline{B}^{2}_{n}}\sum_{i=1}^{n} \bigl\vert \underline{ \sigma }^{2}\cdot \overline{\sigma }_{i}^{2}-\underline{\sigma }_{i} ^{2} \bigr\vert +C_{h}\varepsilon^{\alpha } \\ &{}+\frac{C_{h}}{\overline{B}_{n}^{2}} \sum _{i=1}^{n} \mathbb{E}\bigl[ \vert X_{i} \vert ^{2}I\bigl( \vert X_{i} \vert > \varepsilon \overline{B}_{n}\bigr)\bigr] \\ &{}+C_{h}\Biggl(\frac{1}{\overline{B}_{n}^{2}}\sum_{i=1}^{n} \mathbb{E}\bigl[ \vert X _{i} \vert ^{2}I\bigl( \vert X_{i} \vert > \varepsilon \overline{B}_{n}\bigr)\bigr] \Biggr)^{1+ \frac{\alpha }{2}}. \end{aligned}$$

Thus we obtain (3.1). □

By a similar method, we can obtain a bound on the distance between the normalized sum distribution $\mathbb{E}[\varphi (\frac{S_{n}}{ \underline{B}_{n}})]$ and the corresponding G-normal distribution $\mathbb{E}[\varphi (\eta)]$. And it can also be used to derive the CLT for normalizing factor $\underline{B}_{n}$. We only give the theorem and omit the proof.

### Theorem 3.2

*Let* *η* *be* *G*-*normal distributed on a G*-*expectation space* $(\widetilde{\varOmega },L_{G}^{2}(\widetilde{\varOmega }), \widetilde{\mathbb{E}})$ *with* $\eta \sim \mathcal{N}(\{0\};[1,\overline{ \sigma }^{2}])$, $\overline{\sigma }\geq 1$.

*Then*, *for any fixed* $\varphi \in C_{b,\mathrm{Lip}}(\mathbb{R})$ *and any* $h>0$, $0<\varepsilon <1$, *there exist some* $0<\alpha < 1$, $C>0$, *and* $C_{h}>0$ (*a constant depending on* *h*) *such that*

$$\begin{aligned} & \biggl\vert \mathbb{E}\biggl[\varphi \biggl(\frac{S_{n}}{\underline{B}_{n}}\biggr)\biggr]- \widetilde{\mathbb{E}}\bigl[\varphi (\eta)\bigr] \biggr\vert \\ &\quad \leq \frac{C_{h}}{\underline{B}_{n}^{2}}\sum_{i=1}^{n} \bigl\vert \overline{ \sigma }^{2}\cdot \underline{\sigma }_{i}^{2}-\overline{\sigma }_{i} ^{2} \bigr\vert +\frac{C_{h}}{\underline{B}_{n}^{2}}\sum_{i=1}^{n} \mathbb{E}\bigl[ \vert X _{i} \vert ^{2}I\bigl( \vert X_{i} \vert >\varepsilon \underline{B}_{n}\bigr)\bigr] \\ &\qquad {}+C_{h}\Biggl(\frac{1}{\underline{B}_{n}^{2}}\sum _{i=1}^{n} \mathbb{E}\bigl[ \vert X _{i} \vert ^{2}I\bigl( \vert X_{i} \vert > \varepsilon \underline{B}_{n}\bigr)\bigr]\Biggr)^{1+\frac{\alpha }{2}} \\ &\qquad {}+C_{h} \varepsilon^{\alpha }+C(\sqrt{h}+\sqrt{1+h}-1). \end{aligned}$$

*If we further assume that*

1. $$ \lim_{n\rightarrow \infty }\frac{1}{\underline{B}_{n}^{2}}\sum_{i=1} ^{n} \bigl\vert \overline{\sigma }^{2}\cdot \underline{ \sigma }_{i}^{2}-\overline{ \sigma }_{i}^{2} \bigr\vert =0. $$
2. *For any* $\varepsilon >0$,
  $$ \lim_{n\rightarrow \infty }\frac{1}{\underline{B}_{n}^{2}}\sum_{i=1} ^{n} \mathbb{E}\bigl[ \vert X_{i} \vert ^{2}I \bigl( \vert X_{i} \vert >\varepsilon \underline{B}_{n} \bigr)\bigr]=0. $$

*Then*, *for any* $\varphi \in C_{b,\mathrm{Lip}}(\mathbb{R})$,

$$ \lim_{n\rightarrow \infty }\mathbb{E}\biggl[\varphi \biggl(\frac{S_{n}}{ \underline{B}_{n}} \biggr)\biggr]=\widetilde{\mathbb{E}}\bigl[\varphi (\eta)\bigr]. $$

## Central limit theorem for capacity

The following theorem is the CLT for capacity under the Lindeberg condition.

### Theorem 4.1

*Assume that*

1. *ξ* *is* *G*-*normal distributed on a G*-*expectation space* $(\widetilde{\varOmega },L_{G}^{2}(\widetilde{\varOmega }), \widetilde{\mathbb{E}})$ *with* $\xi \sim \mathcal{N}(\{0\};[\underline{ \sigma }^{2},1])$, $0<\underline{\sigma }\leq 1$, *and*
  4.1 $$ \lim_{n\rightarrow \infty }\frac{1}{\overline{B}_{n}^{2}}\sum_{i=1} ^{n} \bigl\vert \underline{\sigma }^{2}\cdot \overline{ \sigma }_{i}^{2}-\underline{ \sigma }_{i}^{2} \bigr\vert =0. $$
2. *For any* $\varepsilon >0$,
  4.2 $$ \lim_{n\rightarrow \infty }\frac{1}{\overline{B}_{n}^{2}}\sum_{i=1} ^{n} \mathbb{E}\bigl[ \vert X_{i} \vert ^{2}I \bigl( \vert X_{i} \vert >\varepsilon \overline{B}_{n} \bigr)\bigr]=0. $$

*Then*, *for any* $a\in \mathbb{R}$,

4.3 $$ \lim_{n\rightarrow \infty }\mathbb{V}\biggl(\frac{S_{n}}{\overline{B}_{n}} \leq a\biggr)= \widetilde{\mathbb{V}}(\xi \leq a),\qquad \lim_{n\rightarrow \infty }v\biggl( \frac{S_{n}}{\overline{B}_{n}}\leq a\biggr)=\widetilde{v}(\xi \leq a). $$

### Proof

For any fixed $\varepsilon >0$, define

$$ f(x)=\textstyle\begin{cases} 1, &x\leq a, \\ -\frac{1}{\varepsilon }(x-a-\varepsilon),&a< x\leq a+\varepsilon, \\ 0,&x>a+\varepsilon, \end{cases} $$

and

$$ g(x)=\textstyle\begin{cases} 1, &x\leq a-\varepsilon, \\ -\frac{1}{\varepsilon }(x-a), &a-\varepsilon < x\leq a, \\ 0, &x>a. \end{cases} $$

It is easy to verify that $f,g\in C_{b,\mathrm{Lip}}(\mathbb{R})$ and $g(x)\leq I(x\leq a)\leq f(x)$. It follows that

$$\begin{aligned} & \biggl\vert \mathbb{V}\biggl(\frac{S_{n}}{\overline{B}_{n}}\leq a\biggr)- \widetilde{ \mathbb{V}}(\xi \leq a) \biggr\vert \\ &\quad = \biggl\vert \mathbb{E}\biggl[I\biggl(\frac{S_{n}}{\overline{B}_{n}}\leq a\biggr) \biggr]- \widetilde{\mathbb{E}}\bigl[I(\xi \leq a)\bigr] \biggr\vert \\ &\quad \leq \biggl\vert \mathbb{E}\biggl[f\biggl(\frac{S_{n}}{\overline{B}_{n}}\biggr)\biggr]- \widetilde{\mathbb{E}}\bigl[g(\xi)\bigr] \biggr\vert \vee \biggl\vert \mathbb{E}\biggl[g\biggl(\frac{S_{n}}{ \overline{B}_{n}}\biggr)\biggr]-\widetilde{\mathbb{E}} \bigl[f(\xi)\bigr] \biggr\vert \\ &\quad \leq \biggl\{ \biggl\vert \mathbb{E}\biggl[f\biggl(\frac{S_{n}}{\overline{B}_{n}}\biggr) \biggr]- \widetilde{\mathbb{E}}\bigl[f(\xi)\bigr] \biggr\vert + \bigl\vert \widetilde{\mathbb{E}}\bigl[f(\xi)\bigr]- \widetilde{\mathbb{E}}\bigl[g(\xi)\bigr] \bigr\vert \biggr\} \\ &\qquad {}\vee \biggl\{ \biggl\vert \mathbb{E}\biggl[g\biggl(\frac{S_{n}}{\overline{B}_{n}}\biggr) \biggr]- \widetilde{\mathbb{E}}\bigl[g(\xi)\bigr] \biggr\vert + \bigl\vert \widetilde{\mathbb{E}}\bigl[g(\xi)\bigr]- \widetilde{\mathbb{E}}\bigl[f(\xi)\bigr] \bigr\vert \biggr\} \\ &\quad \leq \biggl\vert \mathbb{E}\biggl[f\biggl(\frac{S_{n}}{\overline{B}_{n}}\biggr)\biggr]- \widetilde{\mathbb{E}}\bigl[f(\xi)\bigr] \biggr\vert \vee \biggl\vert \mathbb{E}\biggl[g\biggl(\frac{S_{n}}{ \overline{B}_{n}}\biggr)\biggr]-\widetilde{\mathbb{E}} \bigl[g(\xi)\bigr] \biggr\vert + \widetilde{\mathbb{E}}\bigl[ \bigl\vert f( \xi)-g(\xi) \bigr\vert \bigr]. \end{aligned}$$

By Theorem 2.1 we have

$$ \lim_{n\rightarrow \infty } \biggl\vert \mathbb{E}\biggl[f\biggl( \frac{S_{n}}{\overline{B} _{n}}\biggr)\biggr]-\widetilde{\mathbb{E}}\bigl[f(\xi)\bigr] \biggr\vert =0,\qquad \lim_{n\rightarrow \infty } \biggl\vert \mathbb{E}\biggl[g\biggl( \frac{S_{n}}{\overline{B}_{n}}\biggr)\biggr]- \widetilde{\mathbb{E}}\bigl[g(\xi)\bigr] \biggr\vert =0. $$

Then

$$ \limsup_{n\rightarrow \infty } \biggl\vert \mathbb{V}\biggl( \frac{S_{n}}{\overline{B} _{n}}\leq a\biggr)-\widetilde{\mathbb{V}}(\xi \leq a) \biggr\vert \leq \widetilde{\mathbb{E}}\bigl[ \bigl\vert f(\xi)-g(\xi) \bigr\vert \bigr]. $$

Note that

$$ 0\leq f(x)-g(x)\leq I(a-\varepsilon < x\leq a+\varepsilon), $$

which implies

$$ \widetilde{\mathbb{E}}\bigl[ \bigl\vert f(\xi)-g(\xi) \bigr\vert \bigr]\leq \widetilde{\mathbb{V}}(a- \varepsilon < \xi \leq a+\varepsilon). $$

By the arbitrariness of $\varepsilon >0$ and Lemma 2.2, we have

$$ \lim_{n\rightarrow \infty } \biggl\vert \mathbb{V}\biggl(\frac{S_{n}}{\overline{B}_{n}} \leq a\biggr)-\widetilde{\mathbb{V}}(\xi \leq a) \biggr\vert =0, $$

which implies

$$ \lim_{n\rightarrow \infty }\mathbb{V}\biggl(\frac{S_{n}}{\overline{B}_{n}} \leq a\biggr)= \widetilde{\mathbb{V}}(\xi \leq a). $$

Similarly, we can also obtain

$$ \lim_{n\rightarrow \infty }\mathbb{V}\biggl(\frac{S_{n}}{\overline{B}_{n}} \geq a\biggr)= \widetilde{\mathbb{V}}(\xi \geq a). $$

That is,

$$ \lim_{n\rightarrow \infty }v\biggl(\frac{S_{n}}{\overline{B}_{n}}\leq a\biggr)= \widetilde{v}(\xi \leq a). $$

 □

### Remark 4.1

By a similar method, we can also obtain the CLT for capacity for the normalized sum $S_{n}/\underline{B}_{n}$. We omit the details here.

## Central limit theorem for summability methods

Let $c_{i}(\lambda)$ be continuous functions on $(0,\infty)$ or *λ* only valued in $\mathbb{N}^{*}$. Assume that $0\leq c_{i}( \lambda)\leq 1$ and, for any $\lambda >0$,

$$ \sum_{i=1}^{\infty }c_{i}(\lambda)=1+ \theta (\lambda), $$

where $\lim_{\lambda \rightarrow \infty }\theta (\lambda)=0$. Denote $\overline{B}_{\lambda }^{2}=\sum_{i=1}^{\infty }c_{i}(\lambda)^{2}\overline{ \sigma }_{i}^{2}$, $\underline{B}_{\lambda }^{2}=\sum_{i=1}^{\infty }c _{i}(\lambda)^{2}\underline{\sigma }_{i}^{2}$, where $\overline{B} _{\lambda }^{2}<\infty $ for any $\lambda >0$. Assume that $\mathbb{E}$ is continuous from below and regular, then by Lemma 2.5, $S_{\lambda }:=\sum_{i=1}^{\infty }c_{i}(\lambda)X_{i}$ is well defined q.s. under capacity $\mathbb{V}$.

### Theorem 5.1

*Given a sub*-*linear expectation space* $(\varOmega,\mathcal{H},\mathbb{E})$, $\mathbb{E}$ *is continuous from below and regular*. *Assume that*

1. *ξ* *is* *G*-*normal distributed on a G*-*expectation space* $(\widetilde{\varOmega },L_{G}^{2}(\widetilde{\varOmega }), \widetilde{\mathbb{E}})$ *with* $\xi \sim \mathcal{N}(\{0\};[\underline{ \sigma }^{2},1])$, $0<\underline{\sigma }\leq 1$, *and*
  5.1 $$ \lim_{\lambda \rightarrow \infty }\frac{1}{\overline{B}_{\lambda } ^{2}}\sum_{i=1}^{\infty }c_{i}^{2}( \lambda) \bigl\vert \underline{\sigma }^{2} \cdot \overline{\sigma }_{i}^{2}-\underline{\sigma }_{i}^{2} \bigr\vert =0. $$
2. *For any* $\varepsilon >0$,
  5.2 $$ \lim_{\lambda \rightarrow \infty }\frac{1}{\overline{B}_{\lambda } ^{2}}\sum_{i=1}^{\infty }c_{i}^{2}( \lambda)\mathbb{E}\bigl[ \vert X_{i} \vert ^{2}I\bigl(c _{i}(\lambda) \vert X_{i} \vert >\varepsilon \overline{B}_{\lambda }\bigr)\bigr]=0. $$

*Then*, *for any* $a\in \mathbb{R}$,

5.3 $$ \lim_{\lambda \rightarrow \infty }\mathbb{V}\biggl(\frac{S_{\lambda }}{ \overline{B}_{\lambda }}\leq a\biggr)= \widetilde{\mathbb{V}}(\xi \leq a),\qquad \lim_{\lambda \rightarrow \infty }v\biggl( \frac{S_{\lambda }}{\overline{B} _{\lambda }}\leq a\biggr)=\widetilde{v}(\xi \leq a). $$

### Proof

Denote

$$ S_{\lambda,N}=\sum_{i=1}^{N} c_{i}(\lambda)X_{i},\qquad S^{*}_{\lambda,N}= \sum _{i=N+1}^{\infty }c_{i}( \lambda)X_{i},\qquad \overline{B}_{\lambda,N} ^{2}=\sum _{i=1}^{N} c^{2}_{i}(\lambda) \overline{\sigma }_{i}^{2}. $$

Note that $\lim_{N\rightarrow \infty } \overline{B}_{\lambda,N}^{2}= \overline{B}_{\lambda }^{2}$. For any $k>1$, we can choose *N* sufficiently large such that $\overline{B}_{\lambda,N}^{2}\geq \overline{B}_{\lambda }^{2}/k^{2}$.

For any $a>0$, $t>0$, and $X,Y\in \mathcal{H}$, it holds that

$$ \{X+Y\leq a\}\subset \{X\leq a+t\}\cup \bigl\{ \vert Y \vert \geq t\bigr\} . $$

Then

$$ \mathbb{V}(X+Y\leq a)\leq \mathbb{V}(X\leq a+t)+\mathbb{V}\bigl( \vert Y \vert \geq t\bigr). $$

Hence

$$\begin{aligned} \mathbb{V}\biggl(\frac{S_{\lambda }}{\overline{B}_{\lambda }}\leq a\biggr) &= \mathbb{V}\biggl( \frac{S_{\lambda,N}}{\overline{B}_{\lambda }}+\frac{S^{*} _{\lambda,N}}{\overline{B}_{\lambda }}\leq a\biggr) \\ &\leq \mathbb{V}\biggl(\frac{S_{\lambda,N}}{\overline{B}_{\lambda }} \leq a+t\biggr)+\mathbb{V}\biggl( \frac{ \vert S^{*}_{\lambda,N} \vert }{\overline{B}_{\lambda }}\geq t\biggr) \\ &\leq \mathbb{V}\biggl(\frac{S_{\lambda,N}}{\overline{B}_{\lambda,N}} \leq k(a+t)\biggr)+\mathbb{V}\bigl( \bigl\vert S^{*}_{\lambda,N} \bigr\vert \geq t \overline{B}_{ \lambda }\bigr). \end{aligned}$$

For any $\eta >0$, let

$$ f(x)=\textstyle\begin{cases} 1, &x\leq k(a+t), \\ -\frac{1}{\eta }(x-k(a+t)-\eta),&k(a+t)< x\leq k(a+t)+\eta, \\ 0,&x>k(a+t)+\eta, \end{cases} $$

and

$$ g(x)=\textstyle\begin{cases} 1, &x\leq k(a+t)-\eta, \\ -\frac{1}{\eta }(x-k(a+t)), &k(a+t)-\eta < x\leq k(a+t), \\ 0, &x>k(a+t). \end{cases} $$

It is easy to verify that $f,g\in C_{b,\mathrm{Lip}}(\mathbb{R})$ and $g(x)\leq I(x\leq k(a+t))\leq f(x)$. By the proof process of Theorem 4.1, we have

$$\begin{aligned} &\mathbb{V}\biggl(\frac{S_{\lambda,N}}{\overline{B}_{\lambda,N}}\leq k(a+t)\biggr) \\ &\quad \leq \widetilde{\mathbb{V}}\bigl(\xi \leq k(a+t)\bigr)+ \biggl\vert \mathbb{E}\biggl[f\biggl(\frac{S _{\lambda,N}}{\overline{B}_{\lambda,N}}\biggr)\biggr]-\widetilde{\mathbb{E}} \bigl[f( \xi)\bigr] \biggr\vert \vee \biggl\vert \mathbb{E}\biggl[g\biggl( \frac{S_{\lambda,N}}{\overline{B}_{\lambda,N}}\biggr)\biggr]-\widetilde{\mathbb{E}}\bigl[g(\xi)\bigr] \biggr\vert \\ &\qquad {}+\widetilde{\mathbb{V}}\bigl(k(a+t)-\eta < \xi \leq k(a+t)+\eta\bigr). \end{aligned}$$

By (3.1), for any $0<\varepsilon <1$, we have

$$\begin{aligned} & \biggl\vert \mathbb{E}\biggl[f\biggl(\frac{S_{\lambda,N}}{\overline{B}_{\lambda,N}}\biggr)\biggr]- \widetilde{\mathbb{E}}\bigl[f(\xi)\bigr] \biggr\vert \vee \biggl\vert \mathbb{E}\biggl[g\biggl(\frac{S_{\lambda,N}}{ \overline{B}_{\lambda,N}}\biggr)\biggr]-\widetilde{\mathbb{E}} \bigl[g(\xi)\bigr] \biggr\vert \\ &\quad \leq \frac{C_{h}}{\overline{B}_{\lambda,N}^{2}}\sum_{i=1}^{N} c _{i}^{2}(\lambda) \bigl\vert \underline{\sigma }^{2}\cdot \overline{\sigma } _{i}^{2}-\underline{ \sigma }_{i}^{2} \bigr\vert +\frac{C_{h}}{\overline{B}_{ \lambda,N}^{2}}\sum _{i=1}^{N} c_{i}^{2}( \lambda)\mathbb{E}\bigl[ \vert X_{i} \vert ^{2}I\bigl(c _{i}(\lambda) \vert X_{i} \vert >\varepsilon \overline{B}_{\lambda,N}\bigr)\bigr] \\ &\qquad {}+C_{h}\Biggl(\frac{1}{\overline{B}_{\lambda,N}^{2}}\sum_{i=1}^{N} c_{i} ^{2}(\lambda)\mathbb{E}\bigl[ \vert X_{i} \vert ^{2}I\bigl(c_{i}(\lambda) \vert X_{i} \vert > \varepsilon \overline{B}_{\lambda,N}\bigr)\bigr]\Biggr)^{1+\frac{\alpha }{2}} \\ &\qquad {}+C_{h} \varepsilon^{\alpha }+C(\sqrt{h}+\sqrt{1+h}-1). \end{aligned}$$

Note that $\overline{B}_{\lambda,N}^{2}\geq \overline{B}_{\lambda } ^{2}/k^{2}$, we have

$$\begin{aligned} & \biggl\vert \mathbb{E}\biggl[f\biggl(\frac{S_{\lambda,N}}{\overline{B}_{\lambda,N}}\biggr)\biggr]- \widetilde{\mathbb{E}}\bigl[f(\xi)\bigr] \biggr\vert \vee \biggl\vert \mathbb{E}\biggl[g\biggl(\frac{S_{\lambda,N}}{ \overline{B}_{\lambda,N}}\biggr)\biggr]-\widetilde{\mathbb{E}} \bigl[g(\xi)\bigr] \biggr\vert \\ &\quad \leq \frac{k^{2}C_{h}}{\overline{B}_{\lambda }^{2}}\sum_{i=1}^{ \infty }c_{i}^{2}( \lambda) \bigl\vert \underline{\sigma }^{2}\cdot \overline{ \sigma }_{i}^{2}-\underline{\sigma }_{i}^{2} \bigr\vert +\frac{k^{2}C_{h}}{ \overline{B}_{\lambda }^{2}}\sum_{i=1}^{\infty }c_{i}^{2}( \lambda) \mathbb{E}\biggl[ \vert X_{i} \vert ^{2}I \biggl(c_{i}(\lambda) \vert X_{i} \vert > \frac{\varepsilon }{k} \overline{B}_{\lambda }\biggr)\biggr] \\ &\qquad {}+C_{h}\Biggl(\frac{k^{2}}{\overline{B}_{\lambda }^{2}}\sum_{i=1}^{\infty }c_{i}^{2}( \lambda)\mathbb{E}\biggl[ \vert X_{i} \vert ^{2}I \biggl(c_{i}(\lambda) \vert X_{i} \vert > \frac{ \varepsilon }{k}\overline{B}_{\lambda }\biggr)\biggr]\Biggr)^{1+\frac{\alpha }{2}} \\ &\qquad {}+C _{h}\varepsilon^{\alpha }+C(\sqrt{h}+\sqrt{1+h}-1). \end{aligned}$$

In addition, by Lemma 2.5 we have

$$ \mathbb{E}\bigl[ \bigl\vert S^{*}_{\lambda,N} \bigr\vert ^{2}\bigr]=\mathbb{E}\Biggl[ \Biggl\vert \sum _{i=N+1}^{\infty }c_{i}(\lambda)X_{i} \Biggr\vert ^{2}\Biggr]\leq C\sum_{i=N+1}^{\infty }c_{i}^{2}( \lambda)\mathbb{E}\bigl[ \vert X_{i} \vert ^{2}\bigr] \searrow 0 \quad \text{as } N\rightarrow \infty. $$

Let $t=(\mathbb{E}[|S^{*}_{\lambda,N}|^{2}])^{\frac{1}{3}}$. We have $\lim_{N\rightarrow \infty }t=0$ and

$$ \mathbb{V}\bigl( \bigl\vert S^{*}_{\lambda,N} \bigr\vert \geq t\overline{B}_{\lambda }\bigr)\leq \frac{1}{t ^{2}\overline{B}^{2}_{\lambda }}\mathbb{E}\bigl[ \bigl\vert S^{*}_{\lambda,N} \bigr\vert ^{2}\bigr]= \frac{1}{ \overline{B}^{2}_{\lambda }}\bigl(\mathbb{E}\bigl[ \bigl\vert S^{*}_{\lambda,N} \bigr\vert ^{2}\bigr]\bigr)^{ \frac{1}{3}}\rightarrow 0\quad \text{as } N \rightarrow \infty. $$

So we have

$$\begin{aligned} &\mathbb{V}\biggl(\frac{S_{\lambda }}{\overline{B}_{\lambda }}\leq a\biggr) \\ &\quad \leq\widetilde{\mathbb{V}}\bigl(\xi \leq k(a+t)\bigr)+\widetilde{\mathbb{V}} \bigl(k(a+t)- \eta < \xi \leq k(a+t)+\eta\bigr)+\frac{1}{t^{2}\overline{B}^{2}_{\lambda }}\mathbb{E}\bigl[ \bigl\vert S^{*}_{\lambda,N} \bigr\vert ^{2}\bigr] \\ &\qquad {}+\frac{k^{2}C_{h}}{\overline{B}_{\lambda }^{2}}\sum_{i=1}^{\infty }c _{i}^{2}(\lambda) \bigl\vert \underline{\sigma }^{2}\cdot \overline{\sigma } _{i}^{2}-\underline{ \sigma }_{i}^{2} \bigr\vert +\frac{k^{2}C_{h}}{\overline{B} _{\lambda }^{2}}\sum _{i=1}^{\infty }c_{i}^{2}( \lambda)\mathbb{E}\biggl[ \vert X _{i} \vert ^{2}I \biggl(c_{i}(\lambda) \vert X_{i} \vert > \frac{\varepsilon }{k} \overline{B} _{\lambda }\biggr)\biggr] \\ &\qquad {}+C_{h}\Biggl(\frac{k^{2}}{\overline{B}_{\lambda }^{2}}\sum_{i=1}^{\infty }c_{i}^{2}( \lambda)\mathbb{E}\biggl[ \vert X_{i} \vert ^{2}I \biggl(c_{i}(\lambda) \vert X_{i} \vert > \frac{ \varepsilon }{k}\overline{B}_{\lambda }\biggr)\biggr]\Biggr)^{1+\frac{\alpha }{2}}+C _{h}\varepsilon^{\alpha } \\ &\qquad {}+C(\sqrt{h}+\sqrt{1+h}-1). \end{aligned}$$

By (5.1), (5.2), Lemma 2.1, and Lemma 2.2, letting $t=(\mathbb{E}[|S ^{*}_{\lambda,N}|^{2}])^{\frac{1}{3}}$, $N\rightarrow \infty $, $\lambda \rightarrow \infty $, $\varepsilon \rightarrow 0$, and $\eta \rightarrow 0$ in turn, we have

$$ \limsup_{\lambda \rightarrow \infty }\mathbb{V}\biggl(\frac{S_{\lambda }}{ \overline{B}_{\lambda }}\leq a\biggr) \leq \widetilde{\mathbb{V}}(\xi \leq ka)+C( \sqrt{h}+\sqrt{1+h}-1). $$

By the arbitrariness of $k>1$ and $h>0$, we get

5.4 $$ \limsup_{\lambda \rightarrow \infty }\mathbb{V}\biggl(\frac{S_{\lambda }}{ \overline{B}_{\lambda }}\leq a\biggr) \leq \widetilde{\mathbb{V}}(\xi \leq a). $$

On the other hand, for any $a>0$, $0< t< a$, and $X,Y\in \mathcal{H}$,

$$ \{X+Y\leq a\}\supset \{X\leq a-t\}\backslash \bigl\{ \vert Y \vert \geq t \bigr\} . $$

Then

$$ \mathbb{V}(X+Y\leq a)\geq \mathbb{V}(X\leq a-t)-\mathbb{V}\bigl( \vert Y \vert \geq t\bigr). $$

Hence

$$\begin{aligned} \mathbb{V}\biggl(\frac{S_{\lambda }}{\overline{B}_{\lambda }}\leq a\biggr) &= \mathbb{V}\biggl( \frac{S_{\lambda,N}}{\overline{B}_{\lambda }}+\frac{S^{*} _{\lambda,N}}{\overline{B}_{\lambda }}\leq a\biggr) \\ &\geq \mathbb{V}\biggl(\frac{S_{\lambda,N}}{\overline{B}_{\lambda }} \leq a-t\biggr)-\mathbb{V}\biggl( \frac{ \vert S^{*}_{\lambda,N} \vert }{\overline{B}_{\lambda }}\geq t\biggr) \\ &\geq \mathbb{V}\biggl(\frac{S_{\lambda,N}}{\overline{B}_{\lambda,N}} \leq a-t\biggr)-\mathbb{V}\bigl( \bigl\vert S^{*}_{\lambda,N} \bigr\vert \geq t\overline{B}_{\lambda } \bigr). \end{aligned}$$

By the same method as before, we have

$$\begin{aligned} &\mathbb{V}\biggl(\frac{S_{\lambda }}{\overline{B}_{\lambda }}\leq a\biggr) \\ &\quad \geq\widetilde{\mathbb{V}}(\xi \leq a-t)-\widetilde{\mathbb{V}}(a-t- \eta < \xi \leq a-t+\eta)-\frac{1}{t^{2}\overline{B}^{2}_{\lambda }} \mathbb{E}\bigl[ \bigl\vert S^{*}_{\lambda,N} \bigr\vert ^{2}\bigr] \\ &\qquad {}-\frac{C_{h}}{\overline{B}_{\lambda }^{2}}\sum_{i=1}^{\infty }c_{i} ^{2}(\lambda) \bigl\vert \underline{\sigma }^{2}\cdot \overline{\sigma }_{i} ^{2}-\underline{\sigma }_{i}^{2} \bigr\vert -\frac{C_{h}}{\overline{B}_{\lambda }^{2}}\sum _{i=1}^{\infty }c_{i}^{2}(\lambda) \mathbb{E}\biggl[ \vert X_{i} \vert ^{2}I\biggl(c _{i}(\lambda) \vert X_{i} \vert >\frac{\varepsilon }{k} \overline{B}_{\lambda }\biggr)\biggr] \\ &\qquad {}-C_{h}\Biggl(\frac{1}{\overline{B}_{\lambda }^{2}}\sum_{i=1}^{\infty }c _{i}^{2}(\lambda)\mathbb{E}\biggl[ \vert X_{i} \vert ^{2}I\biggl(c_{i}(\lambda) \vert X_{i} \vert > \frac{ \varepsilon }{k}\overline{B}_{\lambda }\biggr)\biggr] \Biggr)^{1+\frac{\alpha }{2}}\\ &\qquad {}-C _{h}\varepsilon^{\alpha }-C(\sqrt{h}+ \sqrt{1+h}-1). \end{aligned}$$

Letting $t=(\mathbb{E}[|S^{*}_{\lambda,N}|^{2}])^{\frac{1}{3}}$, $N\rightarrow \infty $, $\lambda \rightarrow \infty $, $\varepsilon \rightarrow 0$, and $\eta \rightarrow 0$ in turn, we have

$$ \liminf_{\lambda \rightarrow \infty }\mathbb{V}\biggl(\frac{S_{\lambda }}{ \overline{B}_{\lambda }}\leq a\biggr) \geq \widetilde{\mathbb{V}}(\xi \leq a)-C( \sqrt{h}+\sqrt{1+h}-1). $$

By the arbitrariness of $h>0$, we have

5.5 $$ \liminf_{\lambda \rightarrow \infty }\mathbb{V}\biggl(\frac{S_{\lambda }}{ \overline{B}_{\lambda }}\leq a\biggr) \geq \widetilde{\mathbb{V}}(\xi \leq a). $$

Combining (5.5) with (5.4), we obtain

$$ \lim_{\lambda \rightarrow \infty }\mathbb{V}\biggl(\frac{S_{\lambda }}{ \overline{B}_{\lambda }}\leq a\biggr)= \widetilde{\mathbb{V}}(\xi \leq a). $$

Similarly, we can also have

$$ \lim_{\lambda \rightarrow \infty }\mathbb{V}\biggl(\frac{S_{\lambda }}{ \overline{B}_{\lambda }}\geq a\biggr)= \widetilde{\mathbb{V}}(\xi \geq a). $$

This is equivalent to

$$ \lim_{\lambda \rightarrow \infty }v\biggl(\frac{S_{\lambda }}{\overline{B} _{\lambda }}\leq a\biggr)= \widetilde{v}(\xi \leq a). $$

 □

## References

[1] Artzner P., Delbaen F., Eber J.M., Heath D. (1999). Coherent measures of risk. Math. Finance.

[2] Chen Z., Wu P., Li B. (2013). A strong law of large numbers for non-additive probabilities. Int. J. Approx. Reason..

[3] Choquet G. (1954). Theory of capacities. Ann. Inst. Fourier.

[4] Denis L., Hu M., Peng S. (2011). Function spaces and capacity related to a sublinear expectation: application to G-Brownian motion paths. Potential Anal..

[5] Embrechts P., Maejima M. (1984). The central limit theorem for summability methods of iid random variables. Z. Wahrscheinlichkeitstheor. Verw. Geb..

[6] Freedman A., Sember J. (1981). Densities and summability. Pac. J. Math..

[7] Fridy J.A. (1985). On statistical convergence. Analysis.

[8] Hu C. (2016). A strong law of large numbers for sub-linear expectation under a general moment condition. Stat. Probab. Lett..

[9] Hu F. (2011). Moment bounds for IID sequences under sublinear expectations. Sci. China Math..

[10] Hu F., Zhang D. (2010). Central limit theorem for capacities. C. R. Math..

[11] Hu M., Wang F., Zheng G. (2016). Quasi-continuous random variables and processes under the G-expectation framework. Stoch. Process. Appl..

[12] Hu Z., Zhou L. (2015). Multi-dimensional central limit theorems and laws of large numbers under sublinear expectations. Acta Math. Sin. Engl. Ser..

[13] Li M., Shi Y. (2010). A general central limit theorem under sublinear expectations. Sci. China Math..

[14] Li X. (2015). A central limit theorem for m-dependent random variables under sublinear expectations. Acta Math. Appl. Sin. Engl. Ser..

[15] Peng S. (2007). G-expectation, G-Brownian motion and related stochastic calculus of Itô type. Stochastic Analysis and Applications.

[16] Peng, S.: Law of large numbers and central limit theorem under nonlinear expectations (2007). arXiv preprint math/0702358

[17] Peng S. (2009). Survey on normal distributions, central limit theorem, Brownian motion and the related stochastic calculus under sublinear expectations. Sci. China Ser. A, Math..

[18] Peng, S.: Nonlinear expectations and stochastic calculus under uncertainty (2010). arXiv preprint arXiv:1002.4546

[19] Rokhlin D.B. (2015). Central limit theorem under variance uncertainty. Electron. Commun. Probab..

[20] Wang Q., Su C. (1993). Non-uniform Berry–Essen distance for summability methods with applications. Acta Math. Appl. Sin..

[21] Zhang D., Chen Z. (2014). A weighted central limit theorem under sublinear expectations. Commun. Stat., Theory Methods.

[22] Zhang L. (2016). Exponential inequalities under the sub-linear expectations with applications to laws of the iterated logarithm. Sci. China Math..

[23] Zhang, L.: Lindeberg’s central limit theorems for martingale like sequences under nonlinear expectations (2016). arXiv preprint arXiv:1611.01619

[24] Zhang L. (2016). Rosenthal’s inequalities for independent and negatively dependent random variables under sub-linear expectations with applications. Sci. China Math..

[25] Zhang, L., Lin, J.: Marcinkiewicz’s strong law of large numbers for non-additive expectation (2017). arXiv preprint arXiv:1703.00604

[26] Zhong H., Wu Q. (2017). Complete convergence and complete moment convergence for weighted sums of extended negatively dependent random variables under sub-linear expectation. J. Inequal. Appl..

